# Chiral Cocrystal Solid Solutions, Molecular Complexes, and Salts of *N*-Triphenylacetyl-l-Tyrosine and Diamines

**DOI:** 10.3390/ijms20205004

**Published:** 2019-10-10

**Authors:** Agnieszka Czapik, Maciej Jelecki, Marcin Kwit

**Affiliations:** 1 Faculty of Chemistry, Adam Mickiewicz University, Uniwersytetu Poznańskiego 8, 61-614 Poznań, Polandmarcin.kwit@amu.edu.pl (M.K.); 2 Centre for Advanced Technologies AMU, Uniwersytetu Poznańskiego 10, 61-614 Poznań, Poland

**Keywords:** amino acid, cocrystals, molecular complexes, solid solution, trityl group

## Abstract

The molecular recognition process and the ability to form multicomponent supramolecular systems have been investigated for the amide of triphenylacetic acid and l-tyrosine (*N*-triphenylacetyl-l-tyrosine, TrCOTyr). The presence of several supramolecular synthons within the same amide molecule allows the formation of various multicomponent crystals, where TrCOTyr serves as a chiral host. Isostructural crystals of solvates with methanol and ethanol and a series of binary crystalline molecular complexes with selected organic diamines (1,5-naphthyridine, quinoxaline, 4,4′-bipyridyl, and DABCO) were obtained. The structures of the crystals were planned based on non-covalent interactions (O–H···N or N–H^+^···O^−^ hydrogen bonds) present in a basic structural motif, which is a heterotrimeric building block consisting of two molecules of the host and one molecule of the guest. The complex of TrCOTyr with DABCO is an exception. The anionic dimers built off the TrCOTyr molecules form a supramolecular gutter, with trityl groups located on the edge and filled by DABCO cationic dimers. Whereas most of the racemic mixtures crystallize as racemic crystals or as conglomerates, the additional tests carried out for racemic *N*-triphenylacetyl-tyrosine (*rac*-TrCOTyr) showed that the compound crystallizes as a solid solution of enantiomers.

## 1. Introduction

Molecular recognition and formation of host-guest complexes are fundamentals of supramolecular chemistry [1,2,3,4]. Nowadays, the emphasis is put on designing molecules that are able to interact in a predictable way and provide functional systems in all states of matter. The formation of pre-defined solid-state architectures from smaller, molecular building blocks containing the supramolecular synthons in the molecular structure is the main goal of crystal engineering [5]. Similarly to retrosynthesis, the supramolecular synthons were defined by Desiraju as “structural units within supermolecules that can be formed and/or assembled by known or conceivable synthetic operations involving intermolecular interactions” [6]. Therefore, the proper identification of supramolecular synthons within a given molecular system, their capabilities, and limitations is an important step to understand how the molecules interact and which type(s) of aggregates they may form. The common supramolecular synthons are the carboxylic group and secondary amide functionalities, the latter is responsible e.g., for secondary structure of the proteins [7,8].

Even when considering the relatively long history of the research, the rational design of inclusion (host-guest) compounds is still not a fully explored area [9]. Inclusion compounds are of interest to materials chemistry due to their potential applications for the separation of gases and structural isomers. When the host molecule is an optically active chiral system, the formation of inclusion compounds with racemic guests is often accompanied by enantiodiscrimination. Among the chiral molecules used for this purpose, the natural amino acids are convenient substrates for the synthesis of artificial host molecules [10,11,12,13,14,15,16,17,18,19,20,21,22,23,24,25,26,27,28].

The creation of cocrystals is in the interest of both materials and pharmaceutical sciences. It is known that cocrystallization is one of the methods used in pharmacy in tuning properties of materials [29,30]. One of the challenges in this field is obtaining materials that allow the most optimal use of active substances. It has been proven that cocrystals of API’s (active pharmaceutical ingredient) with free amino acids are more soluble in water, thus bioavailable [31,32]. Free amino acids are not the only substances proved to be efficient in this application. Dipeptides are capable of creating frameworks, which can be applied as containers for volatile drugs. Crystals of L-Val-L-Ala have been applied as vessels for anesthetics [33].

Besides the use in pharmaceutical sciences, cocrystals are a scientific subject in the field of materials chemistry [34,35]. Pseudopeptides based on L-tyrosine are able to form a porous structure, which can absorb gaseous nitrogen [36]. Dipeptides such as di-L-phenylalanine build firm supramolecular systems with a solvent molecule, which are potentially useful in developing new nanomaterials [37]. Organic salts consisting of simple derivatives of L-phenylalanine and achiral carboxylic acids create host-frameworks capable of enantioseparation of sulfoxides and nitriles [38]. Amino acids are also subjects in exploration of the enantiodiscrimination processes utilizing zinc complexes, gold nanoparticles, β-cyclodextrine, and resorcin [4] arenes derivatives [39,40,41,42]. These functional materials can be applied as chiral selectors, which was proven by Carotti et al. in ligand-exchange chromatography utilizing cysteine-based ligand and copper(II) ions [43]. The triphenylmethyl group (trityl, Tr) is often used in synthetic organic chemistry for the protection of reactive hydroxyl, amino, and thiol functionalities [44]. Recently, the usability of the trityl group has been expanded on catalysis and medicinal chemistry [45,46,47]. We and others have shown that the stereodynamic propeller-like trityl group, characterized by *P*- or *M*-helicity (i.e., has the sense of a twist of the phenyl groups) [48,49,50,51,52,53,54], when attached to the permanently chiral inducer can act as a chirality reporter adapting its structure to the chiral environment [46].

In the field of supramolecular chemistry, Tr is used for the construction of molecular machines (e.g., wheel-and-axle molecules, molecular gyroscopes, and compasses) [55]. The trityl group, when present in a chiral molecule, can act as a supramolecular protecting group, especially for the amide N–H hydrogen bond functionality [56,57,58,59,60]. Crystals of enantiomerically pure trityl-containing compounds are often composed of molecules differing in helicity of the Tr group. Mostly, these derivatives form higher-order aggregates of the structure of *pseudo*-centrosymmetric dimers, that is, both monomers are characterized by the same permanent chirality at the stereogenic centers but the opposite helicities of the propeller.

Akazome has shown that in the *N*-tritylamino acids, the inclusion of guest molecules might compensate for the loss of hydrogen bonding [55]. However, in the recent study, Rychlewska and coworkers have demonstrated that the presence of additional functional groups in *N*-triphenylacetylamino acids, which might act as strong hydrogen-bond acceptors, led to the back-activation of the N–H group. This structural feature allowed the formation of various assemblies in the solid-state by *N*-triphenylacetylamino acids with a simultaneous deviation from the propeller-like shape of the trityl moiety [57]. It is worth noting that the poor shape complementarity of some *N*-triphenylacetylamino acids led to the creation of crystals of low density characterized by the presence of small structural voids [57].

**Figure 1:**
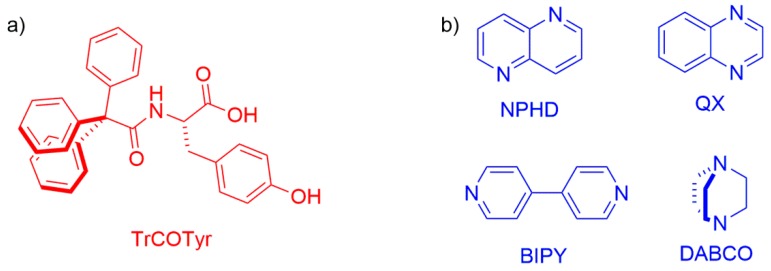
Molecular structure of the compounds under study: (**a**) the host *N*-triphenylacetyl-l-tyrosine molecule (TrCOTyr, red color); (**b**) the guests (blue color): 1,5-naphthyridine (NPHD), quinoxaline (QX), 4,4′-bipyridyl (BIPY), and 1,4-diazabicyclo[2.2.2]octane (DABCO).

Feeling that the problem of the formation of inclusion compounds by trityl-containing amino acids is still at the initial stage of research, we decided to put the emphasis on the tracking of the abilities of selected *N*-triphenylacetylamino acids, namely *N*-triphenylacetyl-l-tyrosine (TrCOTyr, Figure 1), to form inclusion crystalline compounds, as well as tuning the structure of the host molecule in crystal lattice. In this study, we would employ the potentiality of the bulky trityl group to form inclusion compounds additionally enriched by the functionalities present in the parent tyrosine skeleton.

Since there is not much data concerning the abilities of TrCOTyr to form inclusion compounds, we decided to design a number of molecular complexes of TrCOTyr with diamines (Figure 1). Four well-defined and geometrically rigid diamines were selected for the study, namely, 1,5-naphthyridine (NPHD), quinoxaline (QX), 4,4′-bipyridyl (BIPY), and 1,4-diazabicyclo[2.2.2]octane (DABCO). We anticipated that the presence of two amine functionalities in the same molecular skeleton would lead to the assemblies of the 2:1 host-guest ratio.

The previous research on compounds containing trityl groups indicated that these compounds in the crystal tend to form *pseudo*-centrosymmetric systems [58], consisting of heterohelical monomers of the same sense of chirality at the stereogenic center. Therefore, in the course of these studies, we decided to use the racemic form of TrCOTyr as well. It is well-known that racemic compounds can crystallize as racemic crystals (where molecules of enantiomers are distributed in crystal lattice systematically) or as conglomerate (the physical mixture of enantiomericaly pure crystals). The third and most unusual situation is crystallization of a solid solution of enantiomers (called also the mixed crystals), where the enantiomeric molecules in the crystal lattice are arranged statistically. According to literature reports, less than 1% of racemates crystallizes as the solid solution [61] and the phenomenon is a result of a lack of enantioselective intermolecular interactions. Therefore, checking the possibility of forming a solid solution by molecules of *rac*-TrCOTyr, even if statistically unlikely, seemed to us worth some effort.

## 2. Results and Discussion

### 2.1. Polymorphs and Solvates of TrCOTyr·and rac-TrCOTyr

**Figure 2:**
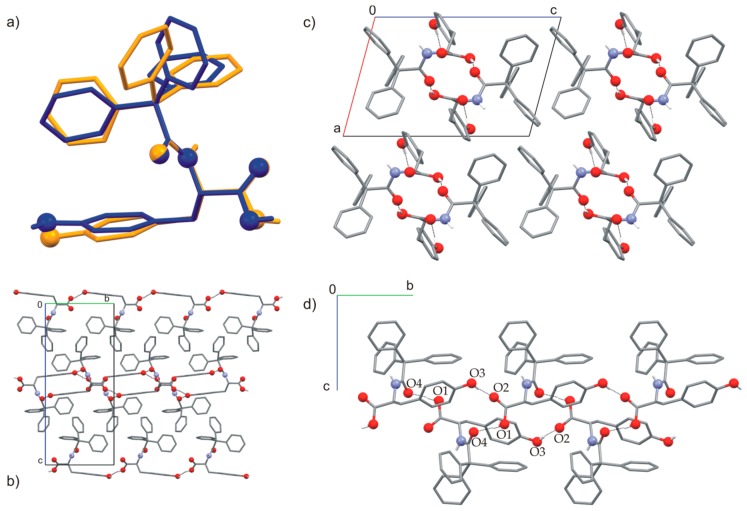
(**a**) Comparison of the molecular conformation of TrCOTyr in polymorph I [42] (orange) and II (blue). Molecular packing in the crystal structure of (**b**) polymorph I, *P*2_1_2_1_2_1_ (view along x-axis) and (**c**) polymorph II, *P*2_1_ (view along y-axis); (**d**) 1D motif observed in the crystal structure of polymorph II. The C-bound hydrogen atoms are omitted for clarity. The O and N atoms are shown as balls.

The crystal structure of TrCOTyr (polymorph I) has been reported recently [42]. The compound crystallizes in the orthorhombic (*P*2_1_2_1_2_1_) space group, where the molecules of TrCOTyr are linked into a 2D motif through two types of O–H···O hydrogen bonds. In the first one of the COO–H···O type, the acceptor is a tyrosine phenyl group. The second one is formed by O–H_phenyl_···O(=C)_amide_ interactions. The carboxylic C=O group does not participate in O–H···O interaction, but is involved in C–H···O interaction. The change of crystallization conditions, involving the replacement of methyl alcohol with isopropyl (or tert-butyl) alcohol, allowed us to obtain a second polymorphic form of TrCOTyr. The second polymorph of TrCOTyr crystallized in a monoclinic (*P*2_1_) space group. In the crystal of polymorph II, molecules of TrCOTyr are bound through O–H···O hydrogen bonds and formed a chain around the 2_1_ axis (Figure 2, Table SI-1). The molecules of TrCOTyr used all available oxygen atoms for the intermolecular interaction, while the NH group is covered by the trityl group, which makes it inaccessible for intermolecular interaction in the crystal lattice. The stability of the supramolecular chains is supported by additional C8–H8···O4^i^ hydrogen bond (Table SI-1).

**Figure 3:**
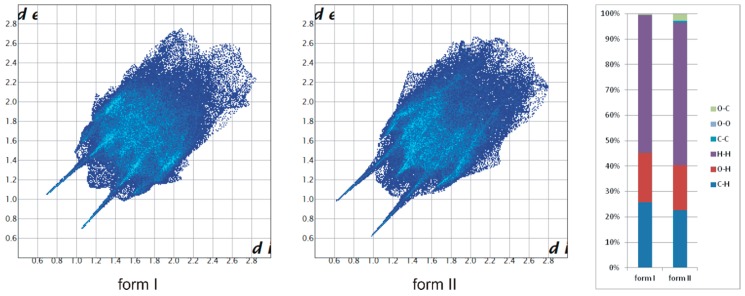
Contribution of intermolecular interactions to the Hirshfeld surface area calculated for polymorphs of TrCOTyr.

The appearance of the new polymorphic form of the compound indicates a certain liability of the molecular conformation. The molecular structure of TrCOTyr in both polymorphic forms was compared (Figure 2a, Table SI-2). In the tyrosine part, the molecule showed high agreement, whereas the conformation of the trityl group changes from *MMM* in polymorph I to *MPM* for polymorph II. Changing the scheme of intermolecular interactions has been traced by analyzing the Hirshfeld surfaces of both polymorphic forms (Figure 3) [62]. Noticeably, for polymorph II, the contribution of the C–O intermolecular interactions increased at the expense of O–H and C–H interactions.

The use of common organic solvents for crystallization experiments provided crystals of either polymorph I or II. However, there are two exceptions. The use of methanol or ethanol for crystallization led to isostructural crystals of solvates TrCOTyr·MeOH and TrCOTyr·EtOH, respectively. In these crystals, TrCOTyr would act as a host, while the function of a guest has been performed by a molecule of the solvent.

**Figure 4:**
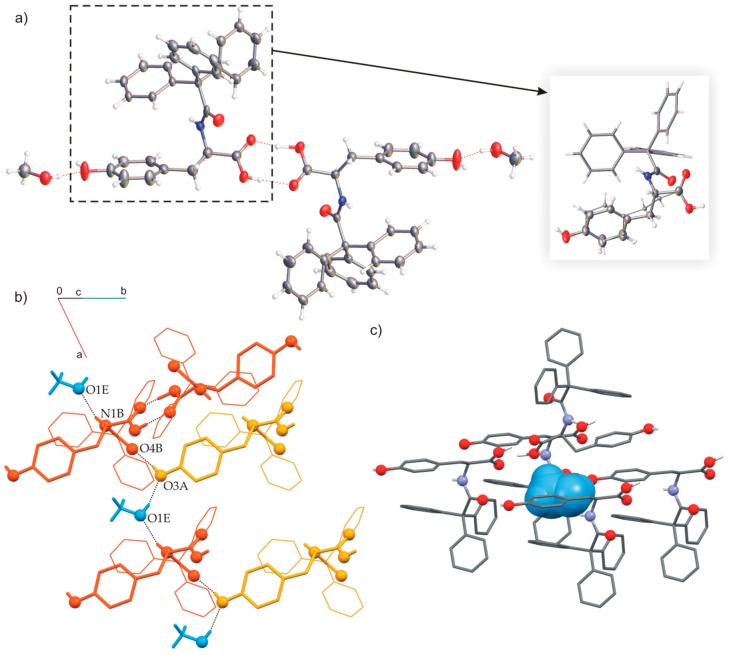
(**a**) The molecular structure of TrCOTyr·MeOH with displacement ellipsoids shown at the 50% probability level, hydrogen bonds are presented as dashed lines (compound crystallize as solid solution of enantiomers – model of disorder for the asymmetric molecule shown in the frame, minor position indicated by thinner bonds); (**b**) 1D motif via hydrogen bonds in crystal structure; (**c**) molecule of methanol trapped between molecules of *N*-triphenylacetyltyrosine.

The molecular structure of TrCOTyr·MeOH is shown in Figure 4. The asymmetric unit of the solvates consists of two independent molecules of *N*-triphenylacetyl-l-tyrosine and two molecules of alcohol (methanol or ethanol, respectively). Amide molecules formed homodimeric, *pseudo*-centrosymmetric building blocks, linked by carboxylic $R_{2}^{2}\left(8\right)$ supramolecular synthon (Figure 4, for hydrogen bonds parameters see Table SI-1).

In the crystal structure, the homodimeric building blocks are linked via hydrogen bonds and the nitrogen atom of the amide group is involved in N–H···O interactions. The molecules of solvent are included in chains of hydrogen bonds and additionally surrounded by molecules of TrCOTyr. As a result, the crystals of solvates turned to become very stable in room conditions.

Careful analysis of the electron differential maps exhibited an additional electron density that was included in the refinement and proved to be a trace of the second enantiomer of the amide, and interestingly, the substitutional disorder appears only on one molecule of amide in homodimers. The *N*-triphenylacetyl-d-tyrosine (*ent*-TrCOTyr) is formed in a small amount during synthesis. The use of l-tyrosine and triphenylacetic acid chloride in an aqueous sodium hydroxide solution led to partial racemization of the resulting amide.

Unexpectedly, the solvates TrCOTyr·MeOH and TrCOTyr·EtOH crystallized as solid solutions of enantiomers. The refined site occupancy indicated 0.92:0.08 and 0.93:0.07 ratio for enantiomers l and d in the solvates with methanol and ethanol, respectively. The HPLC analysis of the samples used for crystallization confirmed the presence of the 8% admixture of *N*-triphenylacetyl-d-tyrosine.

The presence of small amounts of *N*-triphenylacetyl-d-tyrosine and the *pseudo*-centrosymmetric arrangement of the amide molecules in the solvates with methanol and ethanol prompted us to study the crystallization of the intentionally obtained racemic mixture of *N*-triphenylacetyltyrosine (*rac*-TrCOTyr) in more detail.

**Figure 5:**
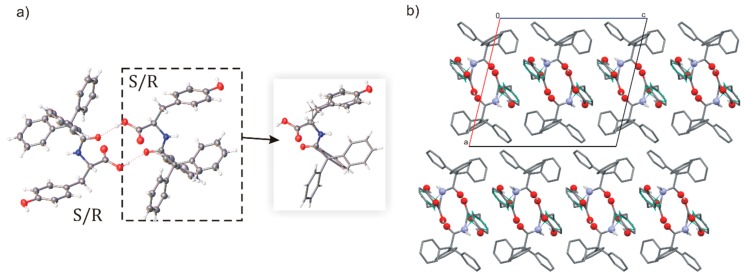
(**a**) Molecular structure of centrosymmetric dimmer in crystal of *rac*-TrCOTyr, hydrogen bonds are presented as dashed lines (compound crystallize as solid solution of enantiomers–model of disorder for the asymmetric molecule is shown in the frame, minor position indicated by thinner bonds) and (**b**) molecular packing in crystal structure.

In the crystal, the *rac*-TrCOTyr molecules formed centrosymmetric dimmers, but carboxylic $R_{2}^{2}\left(8\right)$ supramolecular synthon is not observed (Figure 5). As in the structure of enantiomerically enriched TrCOTyr, all oxygen atoms are involved in O–H···O hydrogen bonds and the NH group does not participate in intermolecular interactions. Interestingly, *rac*-TrCOTyr crystallizes as a solid solution of enantiomers and refined occupancy factors for enantiomers in the measured crystal are 0.90:0.10 (d:l).

Crystals of *rac*-TrCOTyr·MeOH and *rac*-TrCOTyr·EtOH solvates are isostructural and, as expected, they are isostructural with TrCOTyr·MeOH and TrCOTyr·EtOH solvates. The compound crystallized as *P* $\bar{1}$ and the asymmetric unit of solvates *rac*-TrCOTyr·MeOH and *rac*-TrCOTyr·EtOH consisted of one molecule of TrCOTyr and one molecule of methanol or ethanol, respectively. Both *rac*-TrCOTyr·MeOH and *rac*-TrCOTyr·EtOH crystallized as a solid solution of enantiomers and the main building block, the centrosymmetric homodimers, were disordered. The refined occupancy factors showed that the enantiomers ratio in measured crystals is 60:40 (d:l) and 80:20 (d:l), respectively for methanol and ethanol solvate.

### 2.2. Host-Guest Complexes of TrCOTyr

At the next stage of the study, we decided to obtain a series of host-guest complexes of TrCOTyr. We anticipated that the incorporation of a guest molecule between the carboxylic groups would increase the distance between the amide molecules. According to the ‘hydrogen-bond rules’ drafted by Etter in 1991 [63], it was expected that the amine nitrogen atom would be the most preferable hydrogen bond acceptor for the carboxylic group. Thus, the use of diamine guests (shown in Figure 1) would lead to heterotrimeric building blocks.

**Figure 6:**
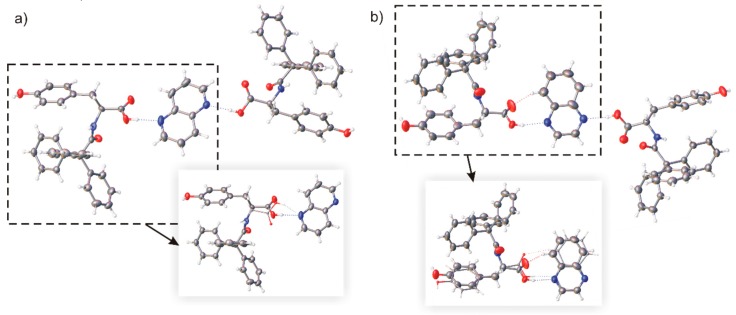
Molecular structure of building blocks in a crystal structure of (**a**) (TrCOTyr)_2_·NPHD and (**b**) (TrCOTyr)_2_·QX; hydrogen bonds are presented as dashed lines, the model of disorder is shown in the frames (minor position indicated by thinner bonds).

The 1,5-naphthyridine molecule is centrosymmetric. This, in principle, should promote the *pseudo*-centrosymmetric arrangement of molecules in trimers. The asymmetric unit cell of co-crystal (TrCOTyr)_2_·NPHD consisted of one molecule of 1,5-naphthyridine and two molecules of *N*-triphenylacetyl-l-tyrosine. Molecules were linked by O–H···N interactions and formed a heterotrimeric basic supramolecular structure, as it was initially planned. In the molecular structure of the heterotrimeric subunit, one molecule of TrCOTyr is slightly disordered in the carboxylic part as a result of rotating the carboxylic group around the C–C bond (Figure 6a) (the refined occupancy factors for both positions are 0.33 and 0.67). Presented dimers are linked into a layer via O–H_phenyl_···O(=C)_amide_ hydrogen bonds with the trityl groups located on the surfaces (Figure SI-2, Table SI-1).

The change of the symmetry of the base would disrupt the symmetry of forming heterotrimers. Crystallization experiments provided for the quinoxaline—TrCOTyr system in all the cases led to obtaining molecular complexes with a 1:1 molar TrCOTyr: QX ratio. In the crystal structure, the main building blocks were formed by a pair of molecules linked by O–H···N hydrogen bond. The asymmetric unit consisted of two such dimers. Despite the change in the structure of the basic supramolecular subunit, *pseudo*-centrosymmetric arrangement of molecules is preserved in the higher-order aggregates and the *pseudo*-center of symmetry is located between the dimers. In the crystal structure, symmetrically dependent dimers are linked via O–H_phenyl_···O(=C)_amide_ interaction in chains and independent chains are ‘glued’ by π···π interaction between molecules of quinoxaline and form a supramolecular tape (Figure SI-3).

To enforce the change in host-guest ratio, the solution of TrCOTyr and QX was nucleated with the crystals of the (TrCOTyr)_2_·NPHD complex. This approach resulted in the formation of a (TrCOTyr)_2_·QX molecular complex, characterized by planned *N*-triphenylacetyl-l-tyrosine: quinoxaline 2:1 ratio. In the molecular structure of heterotrimer, a molecule of quinoxaline and one molecule of amide are disordered (Figure 6b) and the occupancy factors for both positions are 0.5. The crystal structure is a mixture of two kinds of crystals. In the first one, the heterotrimeric building blocks are linked by O–H_phenyl_···O(=C)_amide_ hydrogen bonds into layers similar to those observed in (TrCOTyr)_2_·NPHD (Figure SI-4). In an alternative arrangement observed in crystal, the heterotrimers form supramolecular tapes stabilized by two types of O–H···O hydrogen bonds. The first is, as shown previously, O–H_phenyl_···O(=C)_amide_ interaction and the second is O–H_phenyl_···O=COH hydrogen bond (Table SI-1, Figure SI-3).

**Figure 7:**
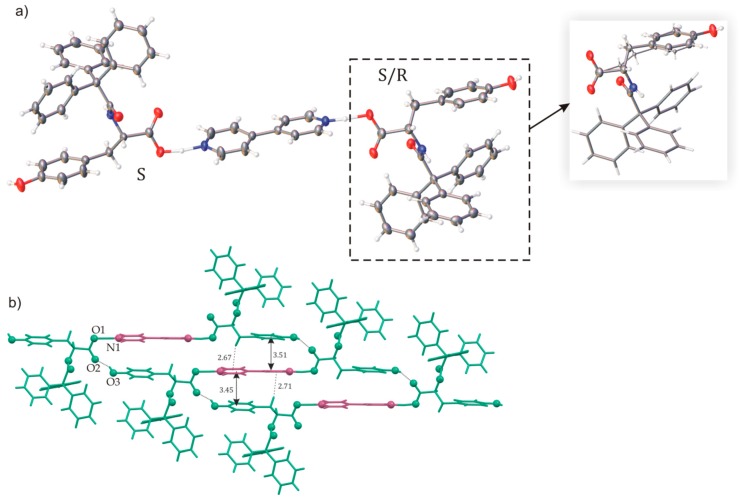
(**a**) Molecular structure of (TrCOTyr)_2_·BIPY (compound crystallizes as solid solution of enantiomers – model of disorder for the asymmetric molecule shown in the frame, minor position indicated by thinner bonds), displacement ellipsoids shown at the 50% probability level; (**b**) molecular tape stabilized by hydrogen bonds and π···π interaction. Distances are in angstroms.

The use of 4,4′-bipyridyl (BIPY) in co-crystallization experiments would lead to an increase in the distance between the host molecules. As it has been expected, the asymmetric unit of the (TrCOTyr)_2_·BIPY co-crystal consisted of the heterotrimeric building block formed by two molecules of TrCOTyr linked by a molecule of 4,4′-bipyridyl (Figure 7a). Careful analysis of the crystallographic data indicated that the molecular complex contained an admixture of about 7.5% of *N*-triphenylacetyl-d-tyrosine and in heterotrimers only one molecule of TrCOTyr was substituted by the second enantiomer.

In the single heterotrimeric (TrCOTyr)_2_·BIPY unit, the donor-acceptor distance was rather short (2.52 Å) and in combination with the slight deformation of the carboxylic group indicated the partial transfer of the protons from the carboxyl groups to the bipyridyl molecule.

Unlike the previously mentioned complexes, in the structure of (TrCOTyr)_2_·BIPY, one could clearly indicate the axle that was formed by BIPY and two wheels, made of the host molecules. The wheel-and-axle-like (TrCOTyr)_2_·BIPY heterotrimeric blocks were bound by O–H···O=C hydrogen- bonding network formed between the OH phenolic group and the carbonyl oxygen atom from adjacent molecules. The tapes thus formed were additionally stabilized by π···π interactions between the bipyridyl and the phenyl ring of the amide molecule (Figure 6). The distances between the aromatic rings were estimated as ranged from 3.45 to 3.51 Å (Figure 7b).

Crystals of *rac*-(TrCOTyr)_2_·BIPY turned out to be isostructural with that of enantiomerically enriched (TrCOTyr)_2_·BIPY crystals. The *rac*-(TrCOTyr)_2_·BIPY also crystallized as a solid solution of enantiomers and the refined occupancy factors indicated that the ratio of *S* and *R* enantiomers in the crystal was 70:30. It is worth emphasizing that the substitutional disorder concerns only one molecule of the amide in the heterotrimer. To date, only one similar example has been reported in the literature— the solid solution of enantiomers in co-crystal of ibuprofen and 4,4′-bipyridyl [64].

Finally, we decided to use aliphatic diamine 1,4-diazabicyclo[2.2.2]octane (DABCO) as the guest molecule. The distinguishing feature of this amine is the considerable basicity of nitrogen atoms.

**Figure 8:**
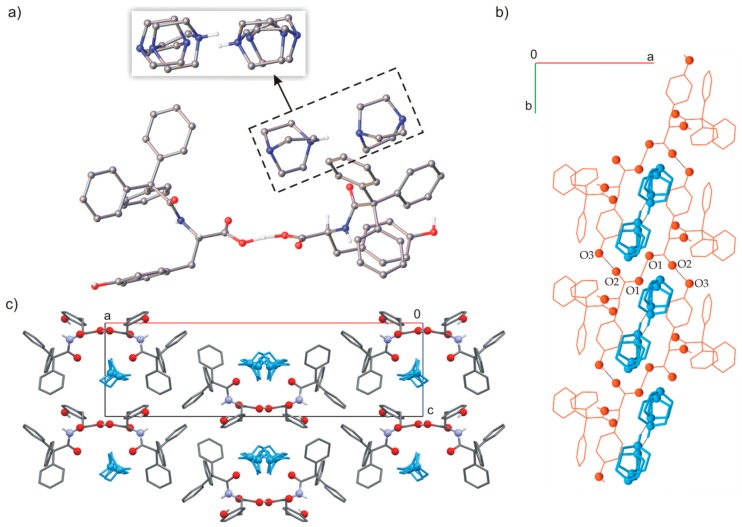
(**a**) Molecular structure of TrCOTyr·DABCO (model of disorder for the DABCO dimer shown in the frame), (**b**) molecular gutter filled by DABCO cationic dimers, (**c**) molecular packing in crystal structure.

The asymmetric unit cell consisted of half of ionic dimer [C_29_H_24_NO_4_]H^−^ and half of disordered ionic DABCO dimer ([C_6_H_12_N_2_]H^+^), both lying on a two-fold axis (Figure 8). The cationic [DABCO]_2_H^+^ was disordered over the 2-fold axis. The TrCOTyr molecules formed ionic dimers, linked through strong, symmetrical O–H···O hydrogen bond (O1A···O1A^i^ 2.425(7) Å; Table SI-1). The anionic dimers formed tapes extended to [010] direction and stabilized by O–H···O interactions. The tape of amides formed supramolecular gutter, with trityl groups located on the edge and filled by DABCO cationic dimers. The position of guest cationic dimers in channels was stabilized by C=O groups directed to the interior of the channels.

Despite many attempts, we were not able to obtain the heterotrimeric structures of the type and architecture that was previously mentioned.

### 2.3. Differential Scanning Calorimetry

**Figure 9:**
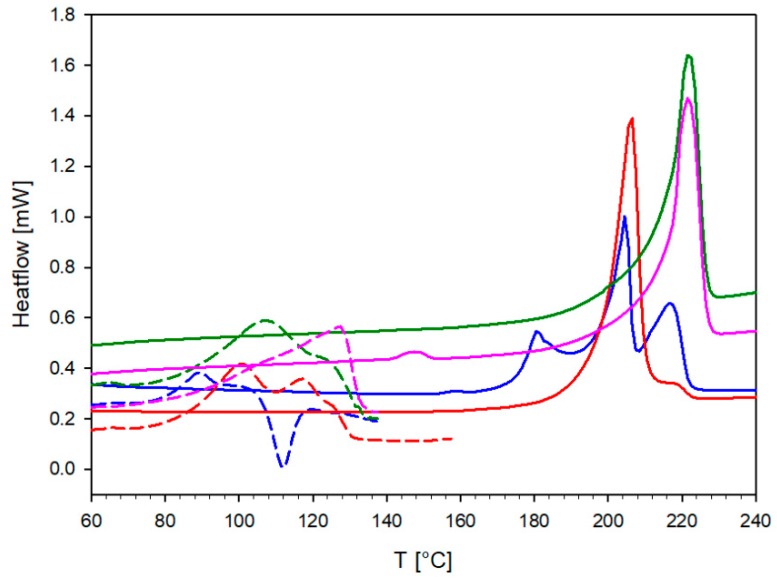
DSC curves of TrCOTyr·MeOH (blue), TrCOTyr·EtOH (red), *rac*-TrCOTyr·MeOH (green), *rac*-TrCOTyr·EtOH (pink). The first heating cycle is shown as dashed lines and the second heating cycle is shown as solid lines.

The DSC analyses were particularly important for characterizing of the solvates. A comparison of DSC curves recorded for them is shown in Figure 9. For all of the investigated samples, in the first heating cycle the melting of a given solvate was observed. For TrCOTyr·MeOH solvate, melting (T_onset_ = 76 °C) and crystallization (T_onset_ = 106 °C) occurred, whereas for TrCOTyr·EtOH solvate, melting was divided into two stages, with two maxima on the DSC curve (peak T_1_ = 101 °C and T_2_ = 118 °C). As expected, for *rac*-TrCOTyr·MeOH and *rac*-TrCOTyr·EtOH solvates, the endothermic peaks were widened, which might occur due to the fact that these solvates were solid solutions of enantiomers (Figure 9).

In the second heating cycle, we observed endothermic peaks corresponding to the melting of the amide. Interestingly, only for TrCOTyr·MeOH after decomposition of the solvate, the remaining amide crystallized as a mixture of both polymorphs. The complex endothermic peak is a set of three transformations—melting of polymorph II of TrCOTyr (T_onset_ = 174 °C), melting of polymorph I (T_onset_ = 199 °C), and peak at T = 216 °C (T_onset_ = 212 °C), which additionally confirms the presence of *rac*-TrCOTyr. That small peak appears also for the measurement carried out for TrCOTyr·EtOH. These conclusions were confirmed by powder measurements of decomposition products (Figure SI-7 and SI-9).

**Figure 10:**
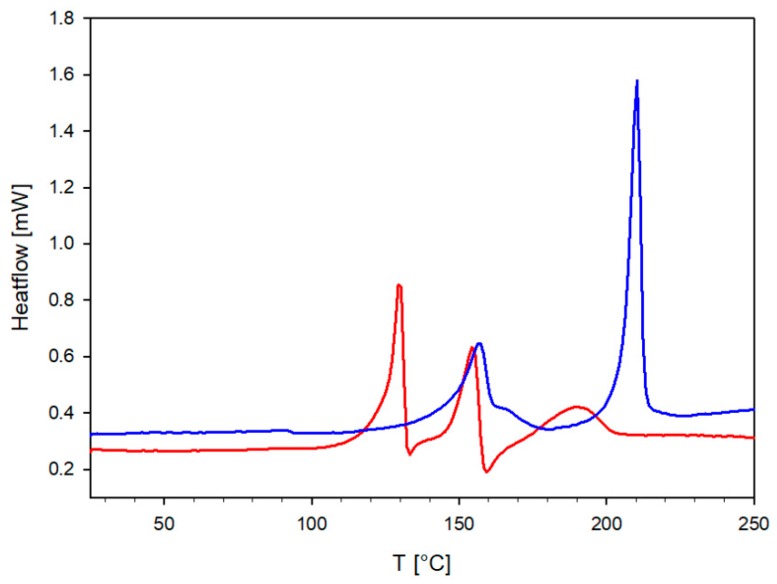
DSC curves of TrCOTyr·QX (red) and (TrCOTyr)_2_·QX (blue).

The DSC curve recorded for TrCOTyr·QX was characterized by a more complex shape (Figure 10). Upon increasing the temperature, melting of the molecular complex TrCOTyr·QX is observed (T_onset_ = 120 °C) and that process is finished with a phase transition (small exothermic peak at T = 133 °C), the formation of (TrCOTyr)_2_·QX is postulated. Another endothermic peak appears at T = 154 °C (T_onset_ = 148 °C) and it stays in agreement with the melting point of (TrCOTyr)_2_·QX appointed in the DSC experiment (T_onset_ = 145 °C). The last broad peak at 190 °C resulted from melting of the polymorph II of TrCOTyr. The second endothermic peak on the DSC curve recorded for the (TrCOTyr)_2_·QX sample is observed at 210 °C (T_onset_ = 205 °C) and corresponds to melting of polymorph I of TrCOTyr.

## 3. Materials and Methods

### 3.1. Synthesis and Crystallization

*N*-Triphenylacetyl-l-tyrosine (and *rac*-*N*-triphenylacetyl-tyrosine) was synthesized according to the previously described procedure [57].

Monocrystals of a new polymorphic form of *N*-triphenylacetyl-l-tyrosine were obtained by slow evaporation of isopropyl alcohol (or *tert*-butyl alcohol) solution to give small colorless crystals. Monocrystals of *rac*-*N*-triphenylacetyl-tyrosine were obtained by slow evaporation of acetone solution to give small colorless plate-shaped crystals, appropriate for X-ray analysis.

Solvates TrCOTyr·MeOH and TrCOTyr·EtOH were obtained by slow evaporation of 1:1 mixture of chloroform and methanol or ethanol. After three days in a microtube, colorless crystals appropriate for X-ray analysis appeared.

Monocrystals of solvates *rac*-TrCOTyr·MeOH and *rac*-TrCOTyr·MeOH were obtained according to the same procedure.

Cocrystals of (TrCOTyr)_2_·NPHD were obtained by dissolving *N*-triphenylacetyl-l-tyrosine and 1,5-naphtirydyne in 2:1 molar ratio in acetone. The solution was allowed to evaporate slowly and crystals suitable for analysis appeared in the vessel after three days.

Cocrystals of TrCOTyr·QX were obtained by dissolving *N*-triphenylacetyl-l-tyrosine and quinoxaline in 2:1 molar ratio in acetone. The solution was allowed to evaporate slowly and crystals suitable for analysis appeared in the vessel after three days. The obtained sample was re-dissolved in acetone and after two days a concentrated mixture was seeded with (TrCOTyr)_2_·NPHD. A day later, crystals of (TrCOTyr)_2_·QX suitable for analysis appeared in the vessel.

Cocrystals (TrCOTyr)_2_·BIPY were obtained by dissolving *N*-triphenylacetyl-l-tyrosine and 4,4’-bipyridyne in 2:1 molar ratio in cold acetone. After two hours, crystals suitable for X-ray analysis appeared in the solution. Cocrystals of (*rac*-TrCOTyr)_2_·BIPY were obtained according to the same procedure.

Crystals of (TrCOTyr)·DABCO were obtained by dissolving *N*-triphenylacetyl-l-tyrosine and DABCO in 1:1 molar ratio in cold acetone. After one hour, tiny crystals, suitable for X-ray analysis, appeared in the solution.

### 3.2. X-Ray Analysis

The diffraction data for monocrystals were collected at 130 K with an Oxford Diffraction SuperNova diffractometer using Co Kα radiation (λ = 1.54184 Å). The intensity data were collected and processed with the use of CrysAlis PRO software (v. 1.171.38.46; Rigaku Oxford Diffraction, Oxfordshire, England). The structures were solved by direct methods with the program SHELXT 2014/7 [65] and refined by full-matrix least-squares method on F^2^ with SHELXL2014/7 [66]. The carbon-bound hydrogen atoms were refined as riding on their carriers and their displacement parameters were set equal to 1.5Ueq(C) for the methyl groups and 1.2Ueq(C) for the remaining H atoms. The hydrogen atoms of OH and NH groups were located in electron-density difference maps. In the final cycles of refinement, they were included in the calculated position and treated as riding atoms. Absolute structures of the compounds were specified by the synthetic procedure and confirmed using Flack parameter [67].

Crystal data, data collection, and structure refinement details are summarized in Table SI-1.

Powder patterns were registered with a four-circle SuperNova diffractometer (Oxford Diffraction, Oxfordshire, England) using Cu K*α* radiation (λ = 1.54184 Å), equipped with mirror monochromator and CCD detector (Atlas, Agilent Technologies, Oxfordshire, England). A 0.3 mm pinhole collimator was used and the detector was set at 70 mm from the sample. The exposure time was fixed to 300 s per scan and images were collected with a 300 degrees phi rotation.

Powder diffractograms have been shown in Figure SI-5–SI-21.

### 3.3. Differential Scanning Calorimetry (DSC)

Melting points were determined using the DSC method in DSC 214 Nevio (NETZSCH, Selb, Germany). Samples were between 6–8 mg, closed in aluminum pans and stored under nitrogen atmosphere (flow rate: 30 mL/min). In the first heating cycle, the heating ramp was set from 25 °C to 160 °C with heating rate 5 °C/min. At this temperature, samples were held for 5 min in isotherm. In the next step, the samples were cooled from 160 °C to 50 °C with the cooling rate of 5 °C/min and then were in constant temperature for 5 min at 50 °C. The last step was heating samples from 50 °C to 250 °C with heating rate 10 °C/min.

Alternatively, the measurements were carried out in one run and the heating ramp was set from 25 °C to 260 °C with heating rate 5 °C/min.

Registered DSC curves have been shown in Figure SI-22–SI-33.

## 4. Conclusions

In this study, we would intend to show the possibility of *N*-triphenylacetyl-tyrosine to form host-guest complexes. It has been found that *N*-triphenylacetyl-tyrosine form crystals that have inclusion properties. The enantiopurity of the starting material is not a prerequisite. The molecules of enantiomerically enriched and racemic *N*-triphenylacetyl-tyrosine form multicomponent inclusion compounds. The potential for hydrogen bonding of the secondary amide groups is well-known, but the NH group of TrCOTyr is active only in crystal structure of solvates with methanol and ethanol. In other structures, we did not observe any hydrogen bonding that involves N–H proton of amide functionality, which confirmed the protecting role of the trityl group in crystal engineering.

There are several types of recognition elements that are involved in guest binding. The most important one is the amide carboxylic group that serves as a donor of the hydrogen bond, whereas the amine nitrogen atoms from the guest molecule serve as an acceptor. This resulted in formation of heterotrimeric basic supramolecular motifs that might form higher-order cyclic structures. In the most interesting case of (TrCOTyr)_2_·BIPY, characterized by the wheel-and-axle structure, the basic heterodimers form tape bound by O–H_phenol_···O=C hydrogen bonding network, further stabilized by π···π interactions between the bipyridyl and the amide phenyl ring of the adjacent molecules. However, the increase of amine basicity led to the formation of ionic species, which should be considered rather as salt, not as host-guest complex.

Solvates of *N*-triphenylacetyl-tyrosine with methanol and ethanol are isostructural, regardless of the optical purity of the host and the type of the solvent. The carboxylic groups form dimeric structures, whereas the phenolic hydroxyl group is a hydrogen bond donor to the solvent molecule. Noticeably, the change of the solvent to a more bulky 2-propanol or *tert*-butanol did not provide respective solvates. Instead, the second polymorph of *N*-triphenylacetyl-l-tyrosine was obtained. In the crystal of polymorph II of TrCOTyr, the monomers are linked by COOH···O=C_amide_ hydrogen bonds.

The presence of a bulky trityl substituent allowed for the formation of a solid solution from racemic mixture of TrCOTyr and its solvates and host-guest complexes. The sterical requirements of the trityl make sense of the chirality at the stereogenic center being of no relevance to the formation of supramolecular assemblies.

## Supplementary Materials

Supplementary materials can be found at https://www.mdpi.com/1422-0067/20/20/5004/s1.

## Abbreviations

Tr	trityl group
TrCOTyr	*N*-triphenylacetyl-l-tyrosine
NPHD	1,5- naphthyridine
QX	quinoxaline
BIPY	4,4′- bipyridyl
DABCO	1,4-diazabicyclo[2.2.2]octane
