# Supplementary material for: Chiral Cocrystal Solid Solutions, Molecular Complexes, and Salts of N-Triphenylacetyl-l-Tyrosine and Diamines
Source: Int J Mol Sci. 2019 Oct 10;20(20):5004. doi: 10.3390/ijms20205004 (PMC6829379; doi:10.3390/ijms20205004)
Supplement: Supplementary file 1 [file ijms-20-05004-s001.pdf]

Supporting Information for

Chiral Cocrystal Solid Solutions, Molecular Complexes, and Salts of *N*-Triphenylacetyl-L-Tyrosine and Diamines

Agnieszka Czapik [1,2,*], Maciej Jelecki [1,2] and Marcin Kwit [1,2,]

[1] Faculty of Chemistry, Adam Mickiewicz University, Uniwersytetu Poznańskiego 8, 61-614 Poznań, Poland.
[2] Centre for Advanced Technologies AMU, Uniwersytetu Poznańskiego 10, 61-614 Poznań, Poland.
* Correspondence: agnieszka.czapik@amu.edu.pl

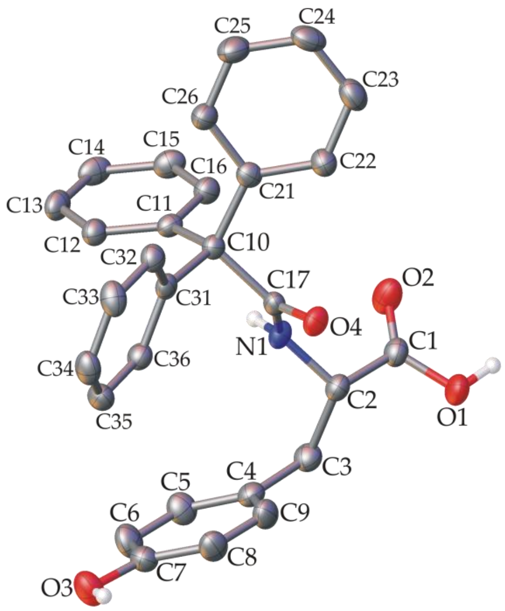

Figure SI-1. Molecular structure of **TrCOTyr**, showing the atom-numbering scheme.

Table SI-1. Geometry of selected hydrogen bonds in crystal structures.

	D—H (Å)	H···*A* (Å)	*D*···*A* (Å)	*D*—H···*A* (°)
**TrCOTyr**				
C8—H8···O4^{i}	0.95	2.51	3.402 (3)	156.6
O1—H1A···O4^{ii}	0.89 (4)	1.72 (4)	2.594 (3)	166 (4)
O3—H3···O2^{iii}	0.90 (4)	1.87 (4)	2.765 (3)	173 (4)
Symmetry code(s): (i) -*x*+1, *y*+1/2, -*z*+1; (ii) -*x*+1, *y*-1/2, -*z*+1; (iii) *x*, *y*+1, *z*.				
**TrCOTyr**·MeOH				
O1A—H1A···O2B	0.98	1.62	2.599 (3)	175.9
O3A—H3A···O4A^{i}	0.82	1.86	2.656 (3)	163.6
N1A—H1AA···O1E^{ii}	0.86	2.15	2.933 (4)	151.8
C3A—H3AA···O1E^{ii}	0.97	2.63	3.350 (4)	131.5
C8A—H8A···O4A^{i}	0.93	2.62	3.282 (4)	128.8
O1B—H1B···O2A	0.91	1.77	2.675 (3)	171.2
O3B—H3B···O4B^{iii}	0.82	1.83	2.634 (4)	165.3
C6A—H6A···O2A^{iii}	0.93	2.69	3.619 (4)	177.8
O1E—H1E···O3A	0.82	2.01	2.781 (4)	157.3
Symmetry code(s): (i) *x*, *y*+1, *z*; (ii) *x*+1, *y*-1, *z*; (iii) *x*, *y*-1, *z*.				
**TrCOTyr**·EtOH				
O1A—H1A···O2B	0.98	1.62	2.593 (3)	172.4
O1B—H1B···O2A	0.98	1.71	2.657 (3)	161.6
O2A—H2A···O1B	0.98	1.69	2.657 (3)	165.0
O2B—H2B···O1A	0.98	1.64	2.593 (3)	163.4
O3A—H3A···O4A^{i}	0.87	1.83	2.692 (3)	173.1
O3B—H3B···O4B^{ii}	0.84	1.84	2.678 (6)	171.5
N1A—H1AA···O1E^{iii}	0.84	2.15	2.943 (3)	155.9
N1B—H1BA···O1F^{iv}	0.98	2.07	2.889 (8)	140.1
N1B—H1BA···O1G^{iv}	0.98	1.86	2.72 (2)	146.1
C3A—H3AA···O1E^{iii}	0.99	2.62	3.353 (4)	130.9
C3A—H3AB···O1F^{v}	0.99	2.64	3.547 (9)	152.8

C6A—H6A···O4A^{i}	0.95	2.55	3.244 (4)	130.0
O1E—H1E···O3A	0.88	1.98	2.852 (4)	169.9
O1F—H1F···O3B	0.84	2.03	2.850 (10)	166.6
C2F—H2FB···O4B^{ii}	0.98	2.67	3.598 (9)	158.3
C2F—H2FC···O1A^{vi}	0.98	2.62	3.591 (11)	170.5
C2G—H2GB···O4B^{ii}	0.98	2.46	3.408 (13)	162.5
C2F—H2FC···O1A^{vi}	0.98	2.63	3.603 (13)	170.4
O1G—H1G···O3B	0.84	2.14	2.94 (3)	159.9
C2G—H2GB···O4B^{ii}	0.98	2.46	3.407 (15)	162.7
Symmetry code(s): (i) *x*, *y*+1, *z*; (ii) *x*, *y*-1, *z*; (iii) *x*+1, *y*-1, *z*; (iv) *x*-1, *y*+1, *z*; (v) *x*-1, *y*+2, *z*; (vi) *x*+1, *y*-2, *z*.				
rac-**TrCOTyr**				
O1—H1···O4^{i}	0.84	1.84	2.671(1)	171.0
O3—H3···O2^{ii}	0.98	1.77	2.732(2)	165.9
C26—H26···O4	0.95	2.26	2.920(2)	125.5
Symmetry code(s): (i) -*x*+1, -*y*, -*z*+1; (ii) *x*, *y*+1, *z*.				
rac-**TrCOTyr**·MeOH				
O1—H1···O2^{i}	1.07	1.58	2.636 (2)	171.6
O3B—H3B···O4^{ii}	0.84	1.73	2.566 (10)	170.3
N1—H1A···O5^{iii}	0.88	2.33	3.129 (7)	150.8
C6—H6···O2^{iv}	0.95	2.64	3.577 (8)	171.1
C8A—H8A···O4^{ii}	0.95	2.55	3.252 (13)	130.4
O3A—H3AA···O4A^{ii}	0.84	1.87	2.706 (14)	178.3
N1A—H1AA···O5^{iii}	0.88	1.90	2.639 (10)	140.7
C2A—H2A···O5^{iii}	1.00	2.59	3.113 (10)	112.5
C6A—H6A···O2^{iv}	0.95	2.55	3.433 (10)	154.4
O5—H5B···O3B	0.84	1.96	2.758 (5)	158.4
O5—H5B···O3A	0.84	2.06	2.846 (6)	155.9
Symmetry code(s): (i) -*x*, -*y*+2, -*z*+1; (ii) *x*, *y*-1, *z*; (iii) *x*-1, *y*+1, *z*; (iv) *x*+1, *y*-1, *z*.				
rac-**TrCOTyr**·EtOH				
O1—H1···O2^{i}	0.98	1.67	2.632 (2)	166.8
O3—H3···O4^{ii}	0.82	1.86	2.677 (6)	178.4
O1B—H1BA···O3	0.82	2.06	2.848 (6)	161.3
O1B—H1BA···O3A	0.82	2.11	2.89 (3)	158.0
O3A—H3AA···O4^{ii}	0.82	1.83	2.55 (3)	146.5
Symmetry code(s): (i) -*x*, -*y*+2, -*z*+1; (ii) *x*, *y*-1, *z*.				
(**TrCOTyr**)$_2$·**NPHD**				
O1A—H1A···N1D	0.82	1.86	2.677 (2)	170.6
O3A—H3A···O4A^{i}	0.82	1.89	2.708 (2)	173.9
O3B—H3B···O4B^{ii}	0.82	1.91	2.698 (2)	161.0
C36B—H36B···O4B	0.93	2.18	2.823 (3)	125.6
O1B—H1B···N5D	0.82	1.87	2.686 (4)	172.8
O1C—H1C···N5D	0.82	1.95	2.744 (7)	162.4
Symmetry code(s): (i) x+1, y-1, z; (ii) x-1, y, z.				
**TrCOTyr·QX**				
O1A—H1A···N1C	0.94	1.79	2.727(3)	171.7
O3A-H3A...O4A^{i}	0.98	1.80	2.778(3)	170.1
O1B-H1B...N1D	0.89	1.81	2.693(3)	172.3
O3B-H3B...O4B^{ii}	0.98	1.87	2.787(3)	153.8
C6B-H6B...O4B^{ii}	0.93	2.59	3.291(4)	132.3
Symmetry code(s): (i) -1+x, y+1, z+1; (ii) 1+x, y, z.				

**(TrCOTyr)$_2$·QX**				
O1A—H1A···N1D	0.82	1.91	2.719 (4)	168.7
O3A—H3A···O4A^{i}	0.82	1.88	2.692 (4)	172.0
C3E—H3E···O3A^{ii}	0.93	2.53	3.420 (19)	159.3
C3E—H3E···O4A^{iii}	0.93	2.54	3.212 (18)	129.6
C5E—H5E···O2C	0.93	2.30	3.190 (12)	161.2
C8E—H8E···O1A	0.93	2.58	3.284 (17)	132.5
O1C—H1C···N4E	0.82	2.00	2.782 (12)	159.0
O1B—H1BA···N4D	0.82	1.98	2.781 (10)	166.2
O3B—H3B···O4B^{iii}	0.82	1.88	2.642 (6)	153.8
C3D—H3D···O3A^{ii}	0.93	2.55	3.35 (2)	145.5
C5D—H5D···O2B	0.93	2.50	3.104 (13)	122.5
Symmetry code(s): (i) x+1, y-1, z; (ii) x-2, y+1, z; (iii) x-1, y, z.				
**(TrCOTyr)$_2$·BIPY**				
N1C—H1B···O1B	1.12 (7)	1.40 (7)	2.519 (4)	176 (6)
O1A—H1A···N2C	1.22 (7)	1.31 (7)	2.517 (5)	169 (5)
O3B—H3BA···O2B^{i}	0.84	1.91	2.734 (4)	167.7
O3A—H3A···O2A^{ii}	0.84	1.96	2.757 (5)	157.9
Symmetry code(s): (i) *x*+1, *y*+1, *z*; (ii) *x*-1, *y*-1, *z*				
(*rac*-**TrCOTyr)$_2$·BIPY**				
N1C—H1A···O1A	1.10 (6)	1.44 (6)	2.523 (6)	164 (5)
O1B—H1B···N2C	1.22 (7)	1.31 (7)	2.516 (6)	169 (6)
O3A—H3A···O2A^{i}	0.84	2.05	2.740 (7)	139.6
O3B—H3B···O2B^{ii}	0.84	1.92	2.734 (7)	164.3
Symmetry code(s): (i) *x*-1, *y*-1, *z*; (ii) *x*+1, *y*+1, *z*				
**TrCOTyr·DABCO**				
O4A—H4A···O3A^{i}	0.82	1.89	2.710 (6)	176.1
N1B—H1B···N1B^{ii}	0.98	1.24	2.11 (4)	144.9
Symmetry code(s): (i) *x*, *y*-1, *z*; (ii) -*x*+1, -*y*+1, *z*				

Table SI-2. Dihedral angles α, β, γ, and ω (in degrees) for **TrCOTyr** molecule observed in the crystal structures of obtained solvates, molecular complexes and salts.

		α[a]	β[b]	γ_1[c]	γ_2[c]	γ_3[c]	ω_1[d]	ω_2[d]	ω_3[d]	
**TrCOTyr** *		-43.6 (5)	9.4	157.40 (15)	34.4 (2)	-80.81 (19)	-54.2 (2)	-18.0 (2)	-66.3 (2)	*MMM*
**TrCOTyr**		-174 (2)	12.8	175.4 (2)	58.5 (3)	-62.4 (2)	-62.8 (3)	10.6 (3)	-55.1 (2)	*MPM*
rac-**TrCOTyr**	S	-150.2	126.5	-177.15 (11)	64.53 (15)	-55.59 (15)	-66.26 (16)	-12.04 (17)	-49.52 (15)	*MMM*
	R	176.6	-56.7							
**TrCOTyr**·MeOH	mol A	-32.0(7)	37.5	-145.6 (3)	92.5 (3)	-25.5 (4)	51.1 (3)	28.0 (4)	56.5 (4)	*PPP*
	mol B	23.4 (7)	168.2	146.5 (3)	26.2 (4)	-92.6 (4)	-54.5 (4)	-54.7 (4)	-23.5 (4)	*MMM*
**TrCOTyr**·EtOH	mol A	-25.7	-82.1	-149.0 (3)	89.6 (4)	-29.4 (4)	46.6 (4)	37.2 (4)	59.8 (4)	*PPP*
	mol B	21.6	62.8	150.3 (3)	29.2 (5)	-90.0 (4)	-51.2 (4)	-57.3 (4)	-34.8 (4)	*MMM*
rac-**TrCOTyr**·MeOH	S	179.8	164.7	143.0 (13)	28 (2)	-85.1 (17)	-48.1 (14)	-53(3)	-27.9 (17)	*MMM*
	R	177.0	-38.7	151.4 (8)	28.9 (13)	-93.2 (10)	-54.3 (8)	-57.6 (19)	-27.0 (10)	*MMM*
rac-**TrCOTyr**·EtOH	S	144.2	147.0	150.57 (17)	29.9 (2)	-88.3 (2)	-47.4 (2)	-58.4 (2)	-36.6 (2)	*MMM*
	R	176.0	-43.7							
(**TrCOTyr**)$_2$·**NPHD**	mol A	-174.9	48.2	-156.3 (2)	82.6 (2)	-34.7 (3)	49.7 (3)	27.8 (3)	61.2 (2)	*PPP*
	mol B	-174.5	31.3	-64.9 (2)	175.59 (18)	54.8 (2)	-62.0 (3)	-36.6 (3)	-41.2 (2)	*MMM*
	mol C	169.8	26.8							
**TrCOTyr·QX**	mol A	49.1 (7)	170.1	157.7 (2)	35.9 (3)	-81.3 (3)	-52.7 (3)	-66.3 (3)	-26.4 (3)	*MMM*
	mol B	-58.4 (9)	35.9	-158.6 (2)	80.6 (3)	-36.3 (3)	51.1 (3)	34.3 (3)	66.6 (3)	*PPP*

	mol A	-176.6	49.2	-154.1 (3)	84.8 (3)	-32.5 (4)	49.1 (4)	29.7 (4)	62.1 (4)	*PPP*
(**TrCOTyr**)$_2$·**QX**	mol B	-179.7	28.7	175.4 (3)	54.5 (4)	-64.6 (4)	-62.9 (4)	-36.5 (5)	-39.2 (4)	*MMM*
	mol B′		18.7							
	A(S)	-170 (3)	23.3	175.8 (3)	57.7 (5)	-63.8 (4)	-60.1 (5)	3.6 (5)	-50.2 (4)	*M0M*
(**TrCOTyr**)$_2$·**BIPY**	B(S)	-171.6	-178.0	-174.0 (3)	65.7 (4)	-55.3 (5)	61.2 (4)	50.9 (4)	-1.0 (5)	*PP0*
	D(R)	-176.3	-23.4							
	A(S)	176.4	23.2	176.0 (5)	57.6 (7)	-64.2 (6)	-59.5 (7)	4.7 (7)	-51.8 (6)	*M0M*
(*rac*-**TrCOTyr**)$_2$·**BIPY**	B(R)	-176.3	-23.3	-175.8 (12)	61.7 (15)	-55.8 (15)	59.0 (9)	58.2 (8)	-8.6 (9)	*PPM*
	D(S)	-174.7	-178.8	-174.1 (17)	67.6 (19)	-56 (2)	62.9 (10)	45.8 (10)	0.7 (11)	*PP0*
**TrCOTyr·DABCO**		177.8	8.9	-178.7 (4)	63.9 (6)	-57.5 (5)	-67.6 (5)	4.3 (7)	-51.8 (5)	*M0M*

[a] – α = O=C-N-H; [a] – β = C-N-C*-H; [c] – γ =O=C-C-C_{ipso}; [d] – ω = (O=)C-C_{Tr}-C_{ipso}-C_{ortho} (of the two possibilities the absolute values ≤90° has been chosen);

* W. Bendzińska-Berus, M. Jelecki, M. Kwit and U. Rychlewska, *CrystEngComm*, 2019, **21**, 3420.

Table SI-3. Selected crystal data and structure refinement details for **TrCOTyr**, *rac*-**TrCOTyr** and received solvates.

	**TrCOTyr**	**TrCOTyr**·MeOH	**TrCOTyr**·EtOH	*rac*-**TrCOTyr**	*rac*-**TrCOTyr**·MeOH	*rac*-**TrCOTyr**·EtOH
Chemical formula	$C_{29}H_{25}NO_4$	$C_{29}H_{25}NO_4 \cdot CH_3OH$	$C_{29}H_{25}NO_4 \cdot C_2H_5OH$	$C_{29}H_{25}NO_4$	$C_{29}H_{25}NO_4 \cdot CH_3OH$	$C_{29}H_{25}NO_4 \cdot C_2H_5OH$
Mr	451.50	483.54	497.57	451.50	483.54	497.57
Crystal system, space group	Monoclinic, $P2_1$	Triclinic, $P1$	Triclinic, $P1$	Monoclinic, $P2_1/c$	Triclinic, $P\bar{1}$	Triclinic, $P\bar{1}$
Temperature (K)	130	130	130	130	130	130
a, *b*, *c* (Å)	8.9457 (2), 10.0804 (2), 13.4851 (3)	8.7715 (2), 9.16082 (19), 17.5443 (4)	8.9324 (3), 9.2946 (2), 17.4235 (5)	14.4519 (3), 10.31245 (19), 16.0925 (4)	8.7284 (4), 9.1575 (4), 17.6624 (8)	8.9189 (2), 9.3109 (4), 17.5130 (7)
α, β, γ (°)	105.031 (2)	79.3563 (18), 81.036 (2), 64.115 (2)	81.032 (2), 82.776 (2), 63.567 (3)	103.605 (2)	79.516 (4), 80.743 (4), 64.146 (4)	81.723 (3), 84.056 (3), 63.479 (3)
V (Å^3)	1174.42 (5)	1241.98 (5)	1276.89 (7)	2331.03 (9)	1243.94 (11)	1286.47 (9)
Z	2	2	2	4	2	2
D_x (Mg m^{-3})	1.277	1.293	1.294	1.287	1.291	1.284
Radiation type	Cu *K*α	Cu *K*α	Cu *K*α	Cu *K*α	Cu *K*α	Cu *K*α
μ (mm^{-1})	0.68	0.71	0.71	0.69	0.71	0.70
Crystal size (mm)	0.4 × 0.2 × 0.05	0.2 × 0.15 × 0.05	0.15 × 0.1 × 0.02	0.2 × 0.1 × 0.05	0.25 × 0.12 × 0.05	0.3 × 0.2 × 0.04
No. of measured, independent and observed [$I > 2\sigma(I)$] reflections	13794, 4309, 4069	44523, 8328, 8117	26357, 8577, 8121	14109, 4214, 3677	24193, 4543, 4077	31528, 5226, 4298
R_{int}	0.035	0.019	0.022	0.028	0.039	0.045
$R[F^2 > 2\sigma(F^2)]$, $wR(F^2)$, S	0.033, 0.084, 1.06	0.038, 0.104, 1.03	0.040, 0.108, 1.08	0.038, 0.101, 1.03	0.059, 0.158, 1.10	0.057, 0.159, 1.04
No. of parameters	316	661	715	363	493	399
$\Delta\rangle_{max}$, $\Delta\rangle_{min}$ (e Å^{-3})	0.14, -0.18	0.42, -0.20	0.50, -0.24	0.37, -0.22	0.22, -0.29	0.34, -0.34
Absolute structure parameter	0.08 (11)	-0.04 (5)	-0.02 (8)			

Table SI-4. Selected crystal data and structure refinement details for molecular complexes of *N*-triphenylacetyl-tyrosine.

	**(TrCOTyr)$_2$·NPHD**	**TrCOTyr·QX**	**(TrCOTyr)$_2$·QX**	**(TrCOTyr)$_2$·BIPY**	**(*rac*-TrCOTyr)$_2$·BIPY**	**TrCOTyr·DABCO**
Chemical formula	$2(C_{29}H_{25}NO_4)\cdot C_8H_6N_2$	$C_{29}H_{25}NO_4\cdot C_8H_6N_2$	$2(C_{29}H_{25}NO_4)\cdot C_8H_6N_2$	$2(C_{29}H_{25}NO_4)\cdot C_{12}H_{10}N_2$	$2(C_{29}H_{25}NO_4)\cdot C_{12}H_{10}N_2$	$[C_{29}H_{24}NO_4]_2H^-$ $\cdot[C_6H_{12}N_2]_2H^+$
Mr	1033.14	581.65	1033.14	1059.18	1059.18	1127.35
Crystal system, space group	Triclinic, *P*1	Triclinic, *P*1	Triclinic, *P*1	Triclinic, *P*1	Triclinic, *P*1	Orthorhombic, $P2_12_12$
Temperature (K)	130	293	130	130	130	130
a, *b*, *c* (Å)	8.8775 (2), 8.9463 (2), 18.4794 (4)	9.16535 (19), 9.41020 (18), 17.7286 (3)	8.8590 (2), 8.9897 (3), 18.5914 (6)	8.9859 (3), 10.2874 (3), 15.8053 (4)	9.1565 (3), 10.3202 (3), 15.7417 (4)	31.7004 (14), 9.9389 (6), 9.3300 (5)
α, β, γ (°)	81.772 (2), 84.236 (2), 66.261 (2)	81.0260 (15), 88.6339 (16), 81.2866 (16)	80.842 (3), 84.908 (2), 66.375 (3)	92.668 (2), 99.587 (2), 111.859 (3)	92.813 (2), 100.097 (3), 112.692 (3)	
V (Å^3)	1328.15 (5)	1492.90 (5)	1338.72 (8)	1327.64 (8)	1339.94 (7)	2939.6 (3)
Z	1	2	1	1	1	2
D_x (Mg m^{-3})	1.292	1.294	1.282	1.325	1.313	1.274
Radiation type	Cu *K*α	Cu *K*α	Cu *K*α	Cu *K*α	Cu *K*α	Cu *K*α
μ (mm^{-1})	0.69	0.68	0.68	0.70	0.69	0.67
Crystal size (mm)	0.3 × 0.25 × 0.17	0.15 × 0.1 × 0.09	0.15 × 0.07 × 0.02	0.05 × 0.05 × 0.02	0.2 × 0.15 × 0.05	0.08 × 0.07 × 0.02
No. of measured, independent and observed [$I > 2\sigma(I)$] reflections	30304, 9248, 8939	30490, 9936, 9705	31450, 9606, 8480	26723, 9214, 7747	28361, 9332, 7942	12275, 5151, 3809
R_{int}	0.020	0.021	0.041	0.043	0.030	0.067
$R[F^2 > 2\sigma(F^2)]$, $wR(F^2)$, *S*	0.029, 0.075, 1.04	0.034, 0.091, 1.03	0.046, 0.133, 1.07	0.041, 0.100, 1.06	0.041, 0.114, 1.07	0.062, 0.164, 1.02
No. of parameters	754	811	871	795	831	370
$\Delta\rangle_{max}$, $\Delta\rangle_{min}$ (e Å^{-3})	0.18, -0.16	0.21, -0.18	0.19, -0.19	0.14, -0.17	0.19, -0.18	0.24, -0.27
Absolute structure parameter	0.04 (5)	-0.06 (7)	0.04 (18)	0.06 (17)	Refined as an inversion twin.	0.4 (3)

Table SI-5. Geometry of caroboxylic groups of **TrCOTyr** molecule.

		O1—C1 (Å)	O2—C1 (Å)	O2—C1—O1 (º)
**TrCOTyr**		1.315 (3)	1.213 (3)	124.9 (2)
**TrCOTyr**·MeOH	A	1.304 (4)	1.223 (4)	111.8 (3)
	B	1.304 (4)	1.212 (4)	124.4 (3)
**TrCOTyr**·EtOH	A	1.245 (4)	1.269 (4)	124.3 (3)
	B	1.264 (4)	1.266 (4)	124.4 (3)
rac-**TrCOTyr**		1.310 (2)	1.205 (2)	125.2 (1)
rac-**TrCOTyr**·MeOH		1.292 (3)	1.221 (3)	124.4 (2)
rac-**TrCOTyr·Et**OH		1.286 (3)	1.239 (3)	124.4 (2)
(**TrCOTyr**)$_2$·**NPHD**	A	1.322 (3)	1.201 (3)	125.5 (2)
	B	1.317 (6)	1.197 (6)	125.2 (4)
	C	1.318 (2)	1.206 (2)	124.2 (1)
**TrCOTyr·QX**	A	1.319 (3)	1.190 (3)	124.4 (3)
	B	1.293 (4)	1.190 (3)	123.5 (3)
(**TrCOTyr**)$_2$·**QX**	A	1.313 (5)	1.202 (5)	124.9 (3)
	B	1.332 (18)	1.20 (2)	124.9 (1)
	C	1.355 (18)	1.17 (2)	123(2)
(**TrCOTyr**)$_2$·**BIPY**	A	1.291 (5)	1.236 (5)	125.5 (4)
	B	1.283 (5)	1.220 (5)	126.8 (4)
(*rac*-**TrCOTyr**)$_2$·**BIPY**	A	1.276 (8)	1.223 (7)	127.1 (5)
	B	1.288 (8)	1.237 (8)	125.6 (6)
**TrCOTyr·DABCO**		1.295 (6)	1.220 (7)	125.3 (5)

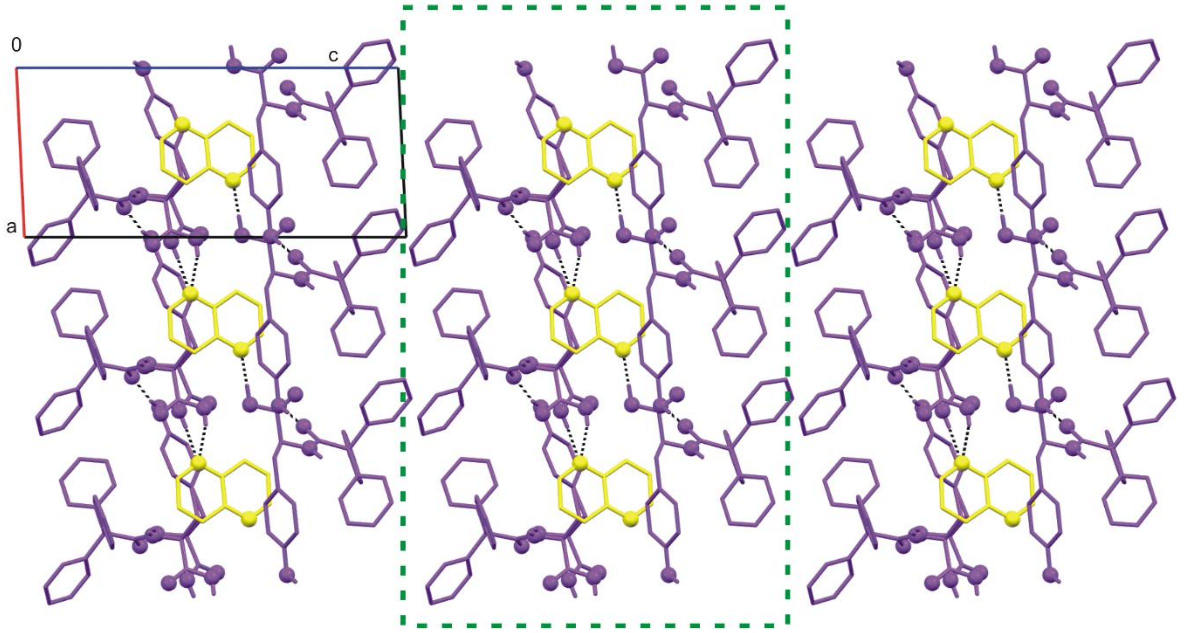

Figure SI-2. Molecular packing in crystal structure of (**TrCOTyr**)$_2$·**QX** (view along y axis), molecular layer is highlited by the frame.

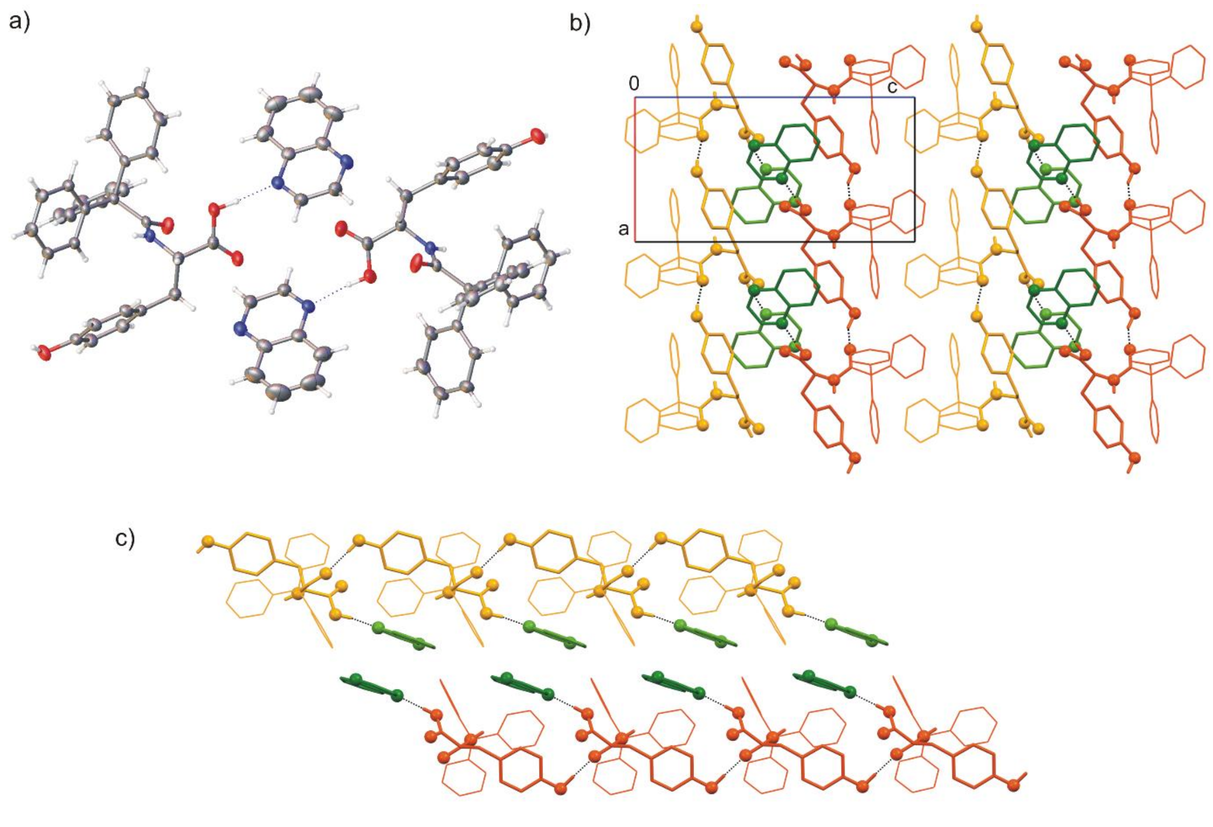

Figure SI-3. (a) Molecular structure of **TrCOTyr·QX**, (b) molecular packing in crystal structure (view along y axis); (c) supramolecular chains glued by π···π interaction (O and N atoms are shown as balls)

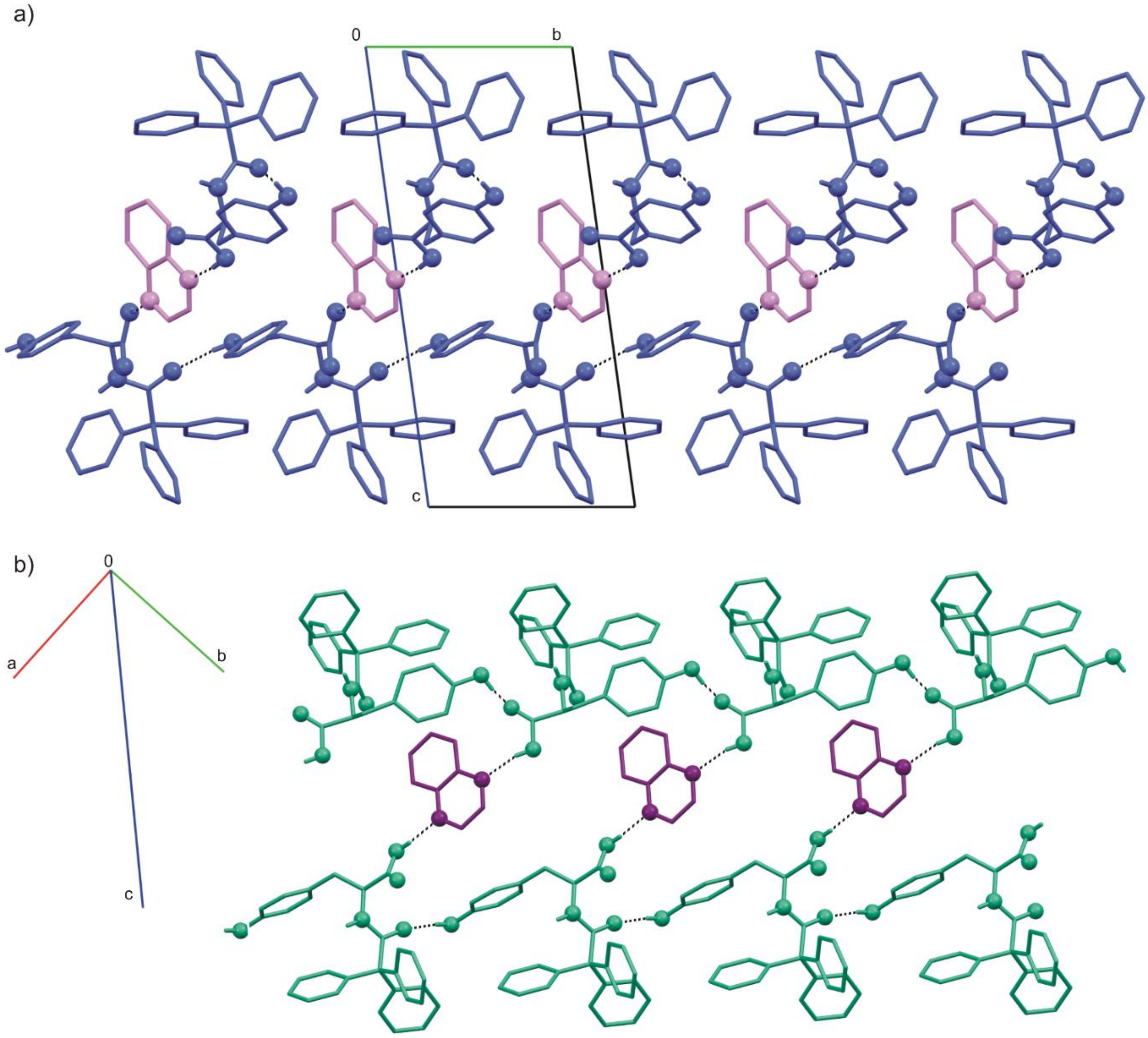

Figure SI-4. Alternative molecular arrangement in crystal structure of (**TrCOTyr**)$_2$·**QX**: (a) molecular layer (b) molecular chain (O and N atoms are shown as balls).

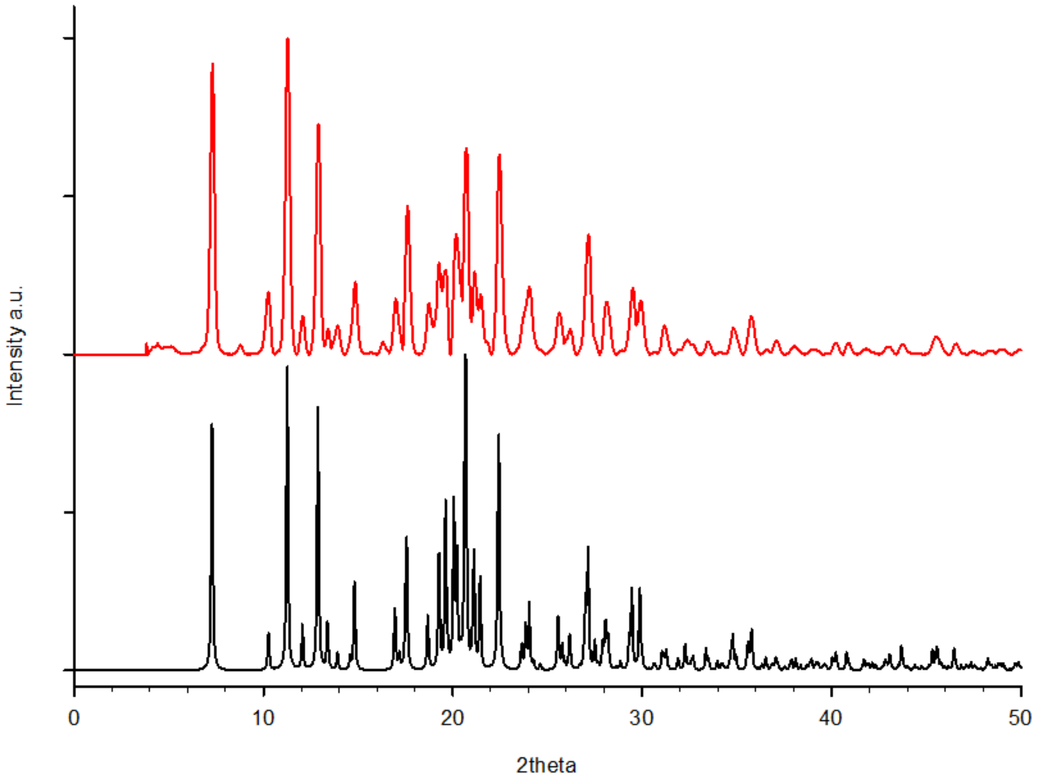

Figure SI-5. The recorded X-ray powder diffraction pattern (red) and predicted pattern based on single-crystal X-ray diffraction indices (black) of **TrCOTyr** (polymorph I).

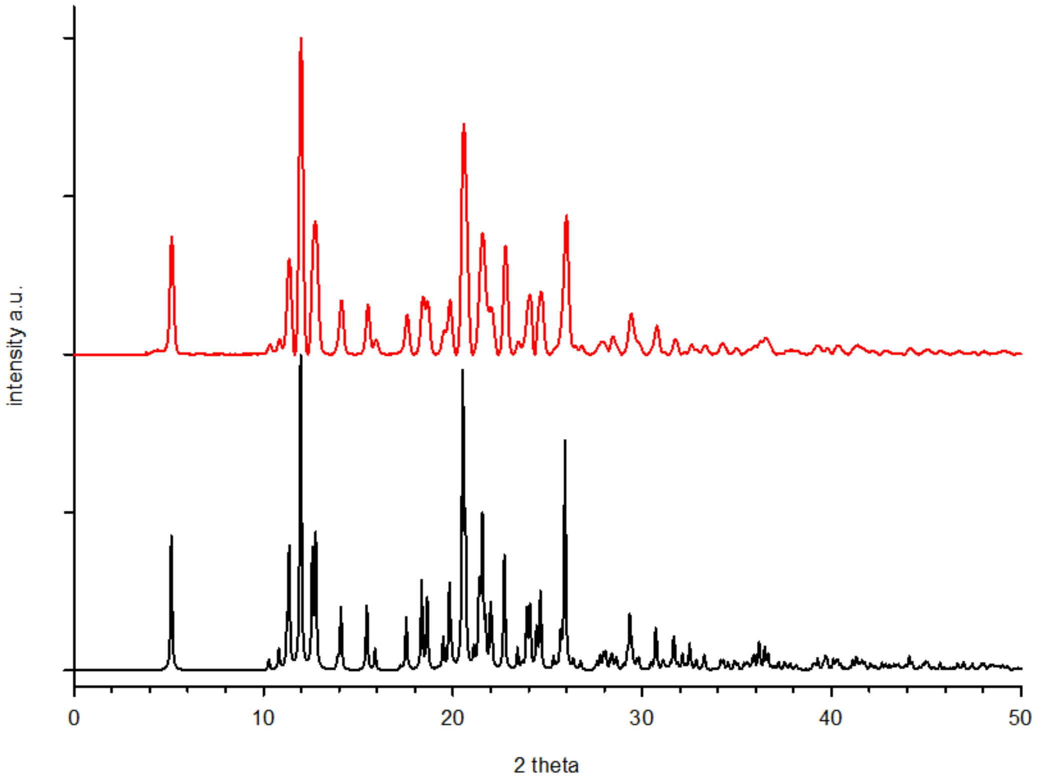

Figure SI-6. The recorded X-ray powder diffraction pattern (red) and predicted pattern based on single-crystal X-ray diffraction indices (black) of **TrCOTyr**·MeOH.

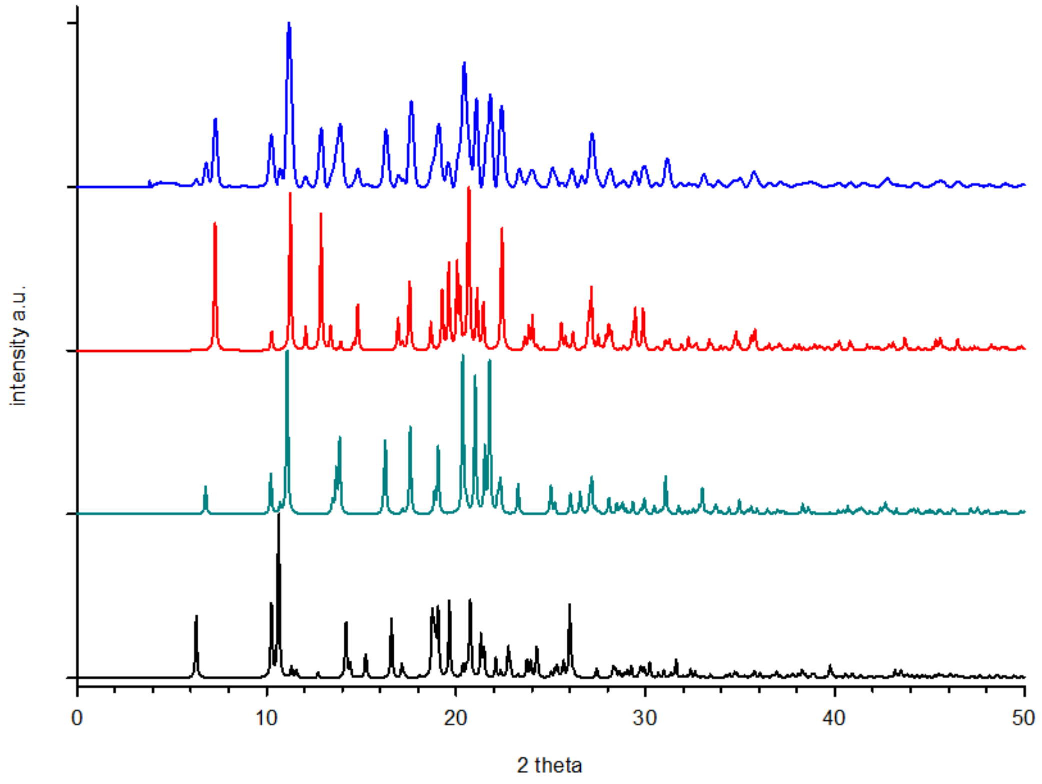

Figure SI-7. The recorded X-ray powder diffraction pattern of decomposition product of **TrCOTyr**·MeOH (blue) and predicted patterns based on single-crystal X-ray diffraction indices of: **TrCOTyr** polymorph I (red), **TrCOTyr** polymorph II (green), *rac*-**TrCOTyr** (black).

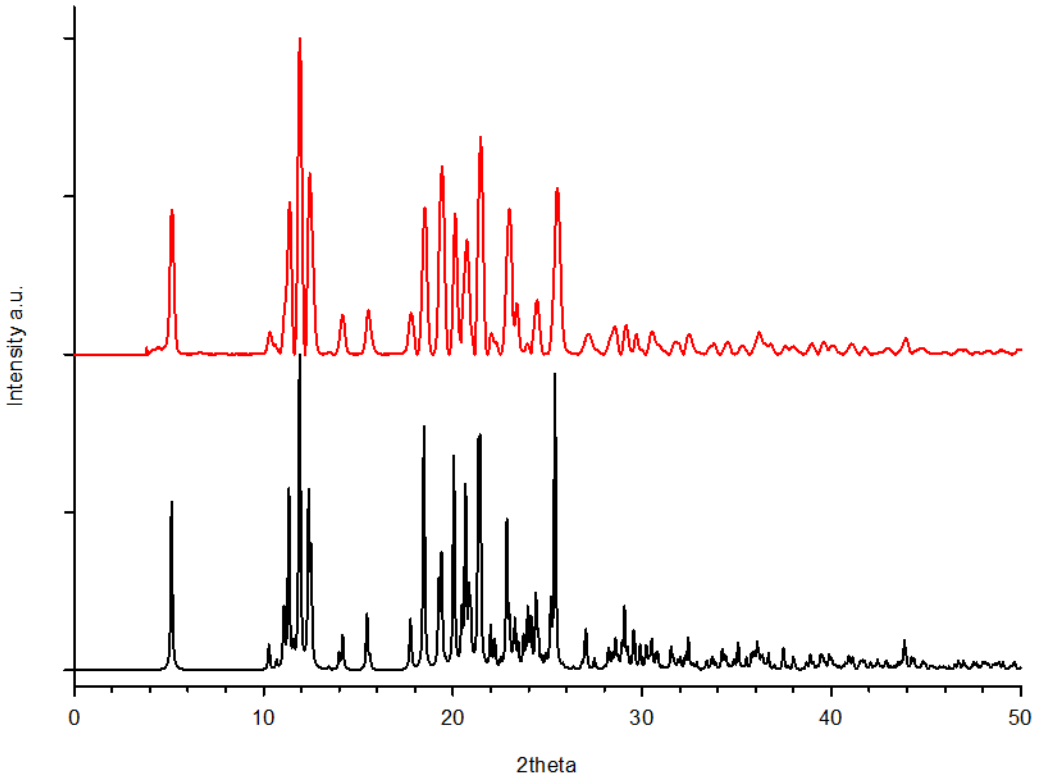

Figure SI-8. The recorded X-ray powder diffraction pattern (red) and predicted pattern based on single-crystal X-ray diffraction indices (black) of **TrCOTyr**·EtOH.

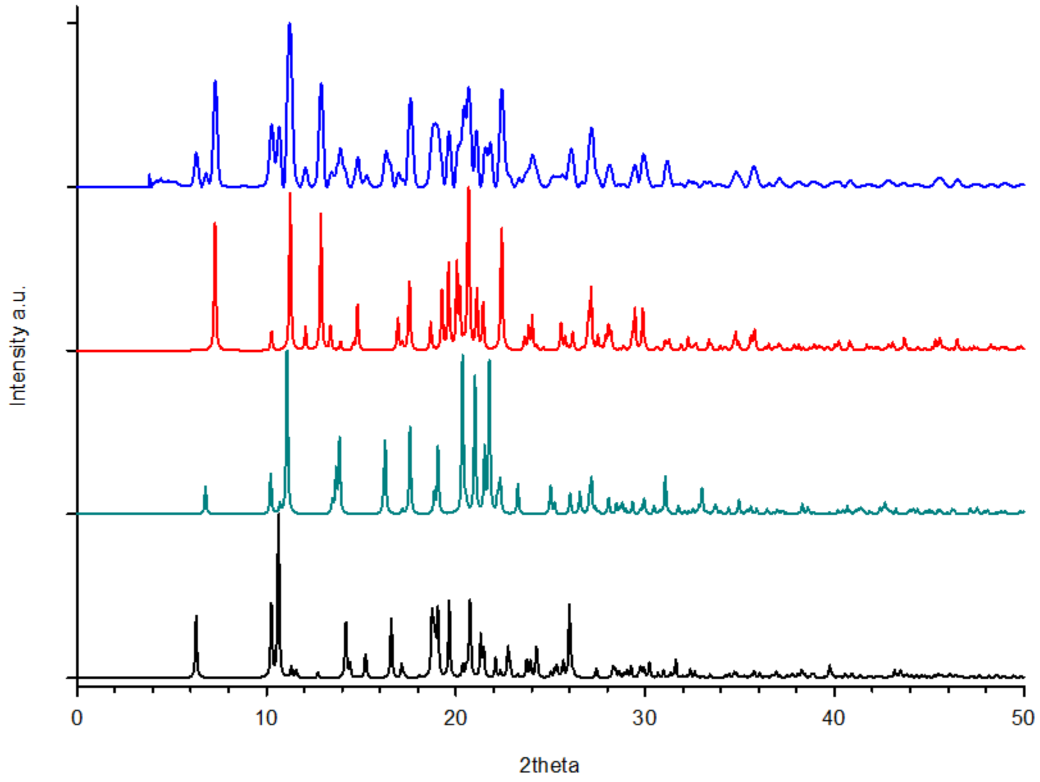

Figure SI-9. The recorded X-ray powder diffraction pattern of decomposition product of **TrCOTyr**·EtOH (blue) and predicted patterns based on single-crystal X-ray diffraction indices of: **TrCOTyr** polymorph I (red), **TrCOTyr** polymorph II (green), *rac*-**TrCOTyr** (black).

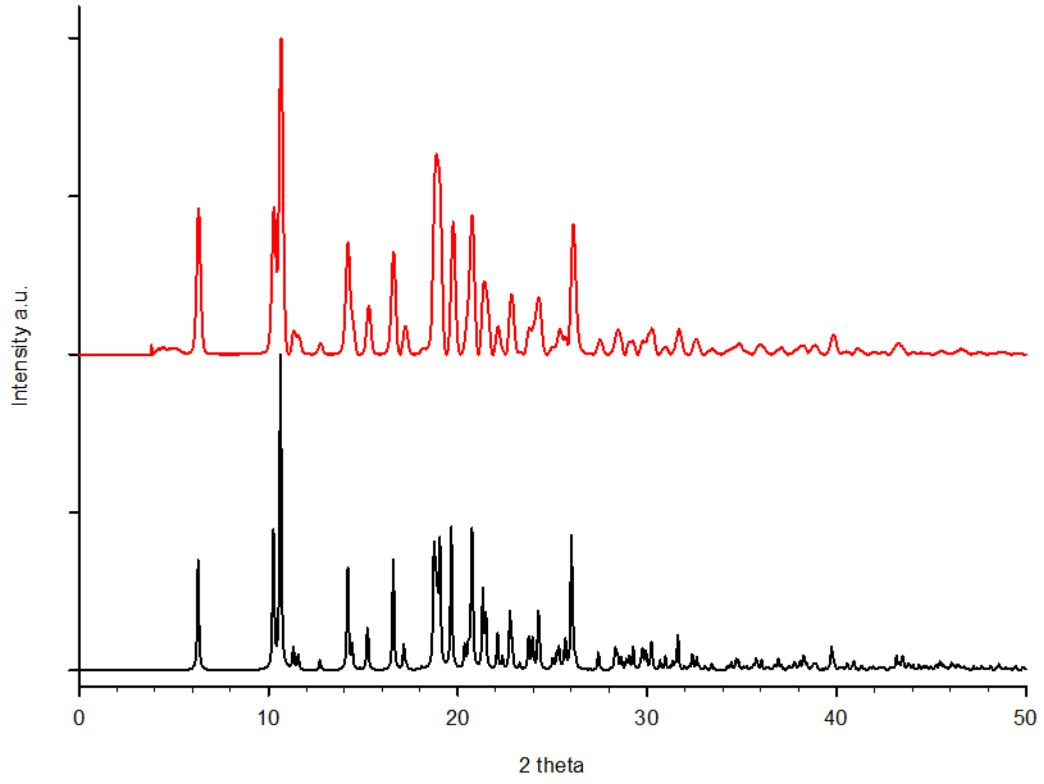

Figure SI-10. The recorded X-ray powder diffraction pattern (red) and predicted pattern based on single-crystal X-ray diffraction indices (black) of *rac*-**TrCOTyr**.

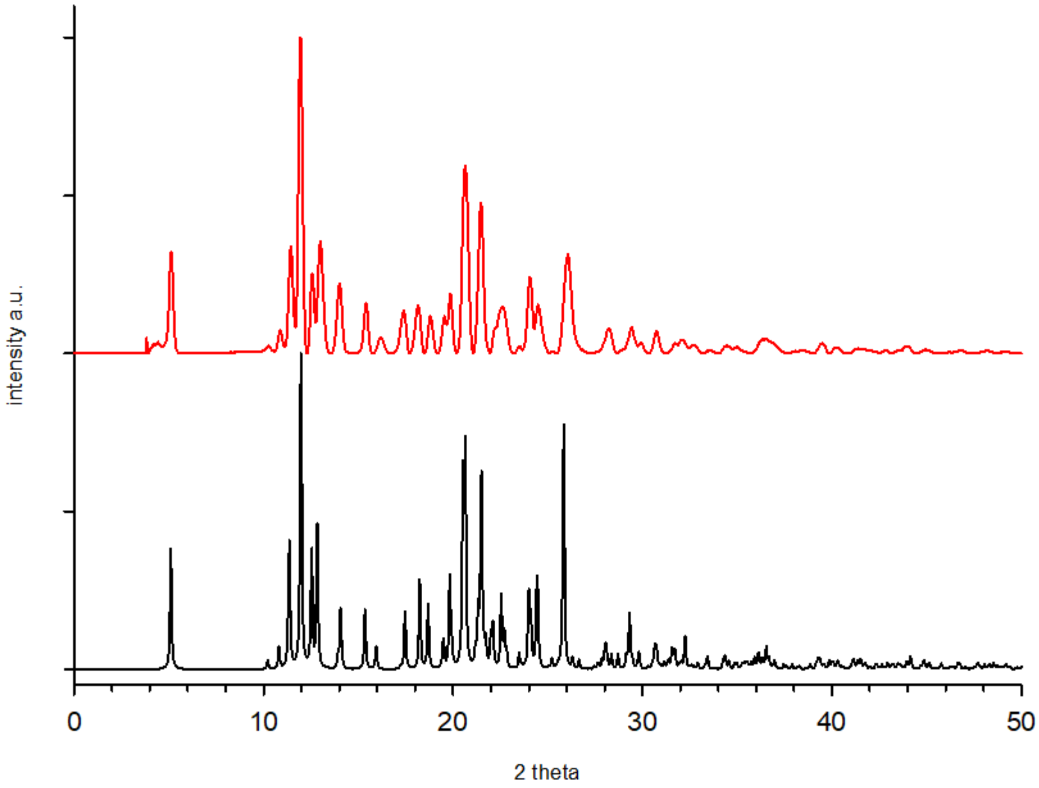

Figure SI-11. The recorded X-ray powder diffraction pattern (red) and predicted pattern based on single-crystal X-ray diffraction indices (black) of *rac*-**TrCOTyr**·MeOH.

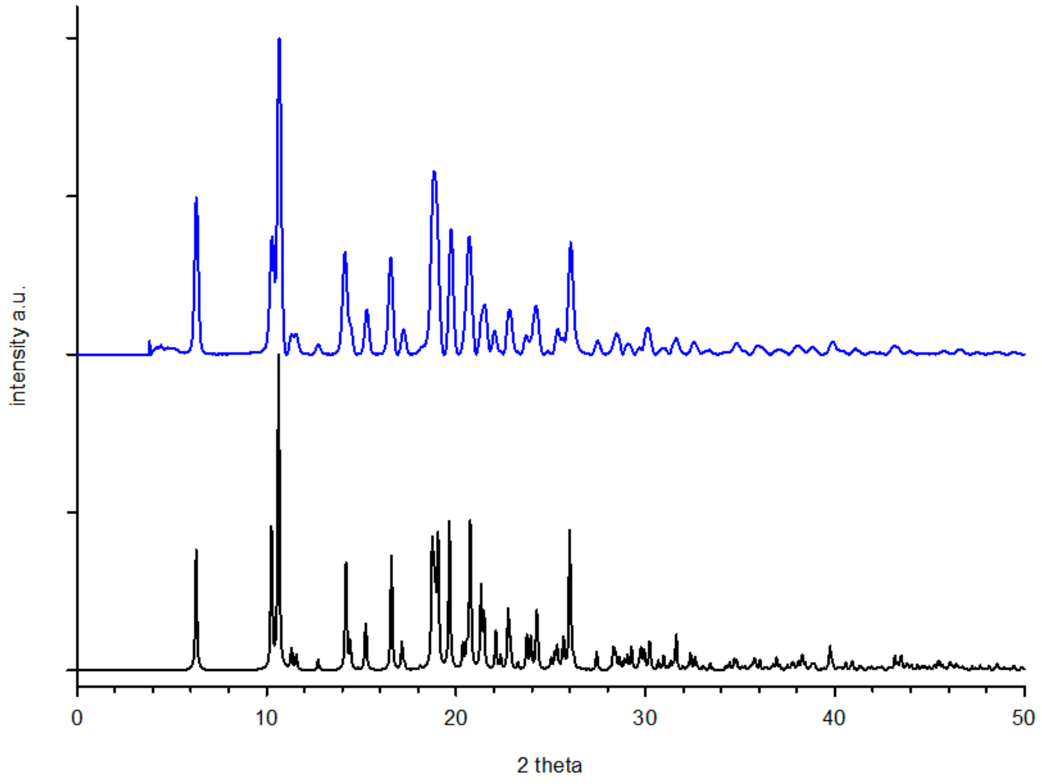

Figure SI-12. The recorded X-ray powder diffraction pattern of decomposition product of *rac*-**TrCOTyr**·MeOH (blue) and predicted patterns based on single-crystal X-ray diffraction indices of *rac*-**TrCOTyr** (black).

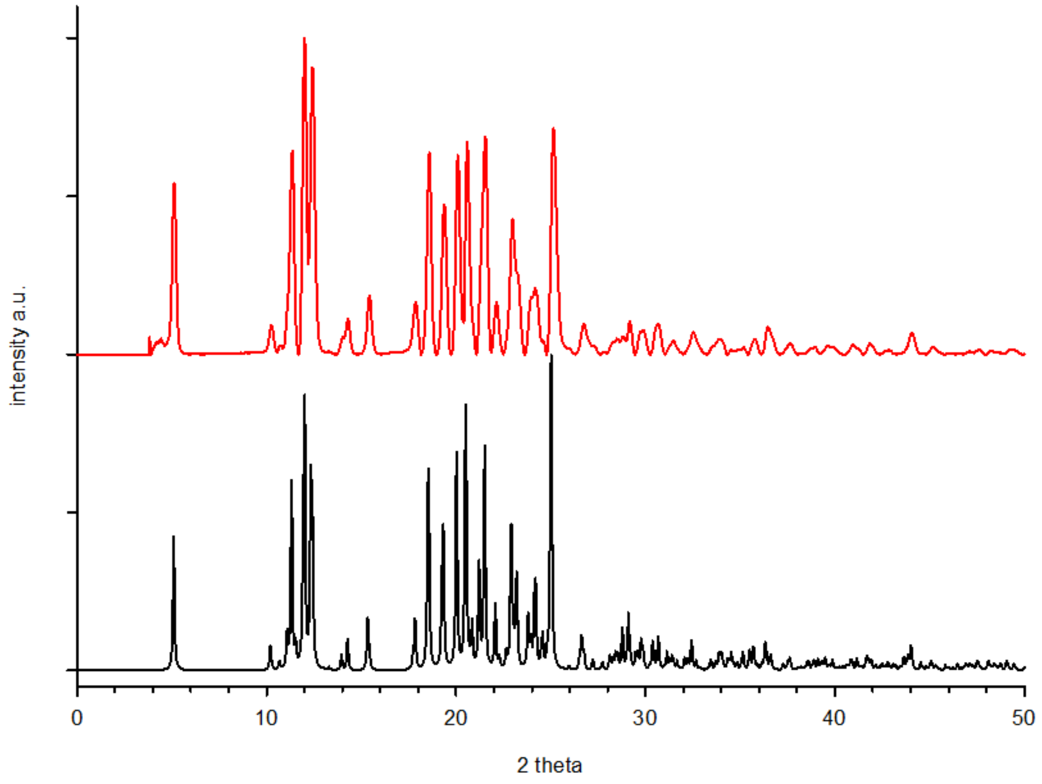

Figure SI-13. The recorded X-ray powder diffraction pattern (red) and predicted pattern based on single-crystal X-ray diffraction indices (black) of *rac*-**TrCOTyr**·EtOH.

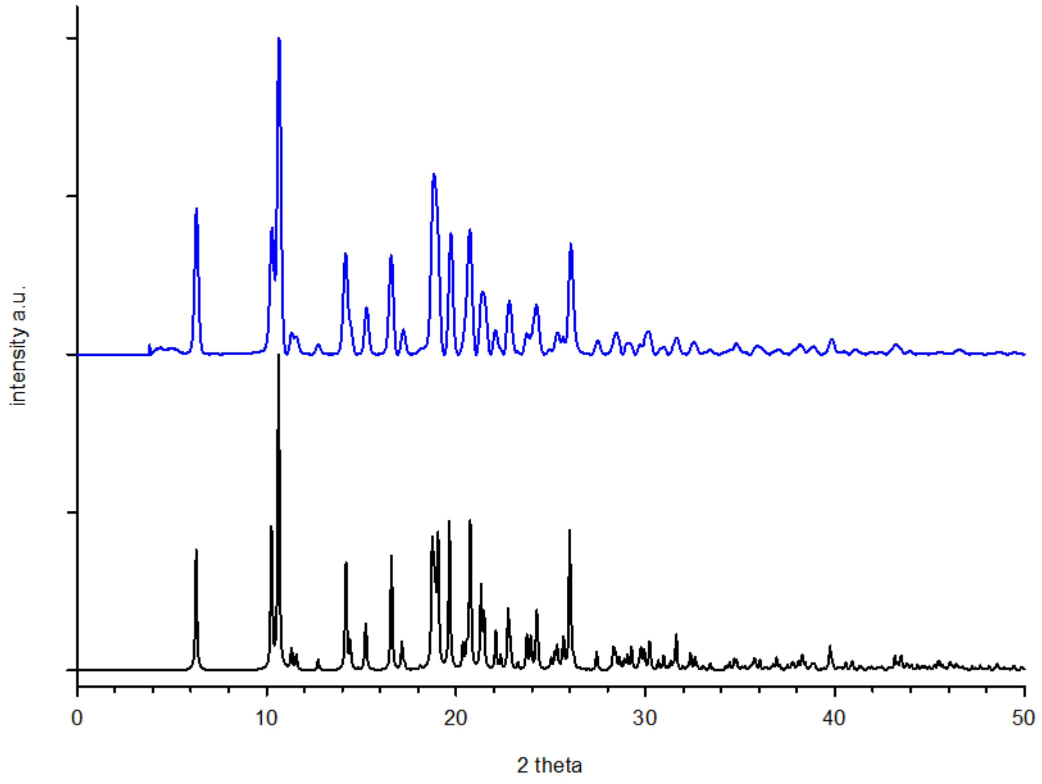

Figure SI-14. The recorded X-ray powder diffraction pattern of decomposition product of *rac*-**TrCOTyr**·EtOH (blue) and predicted patterns based on single-crystal X-ray diffraction indices of *rac*-**TrCOTyr** (black).

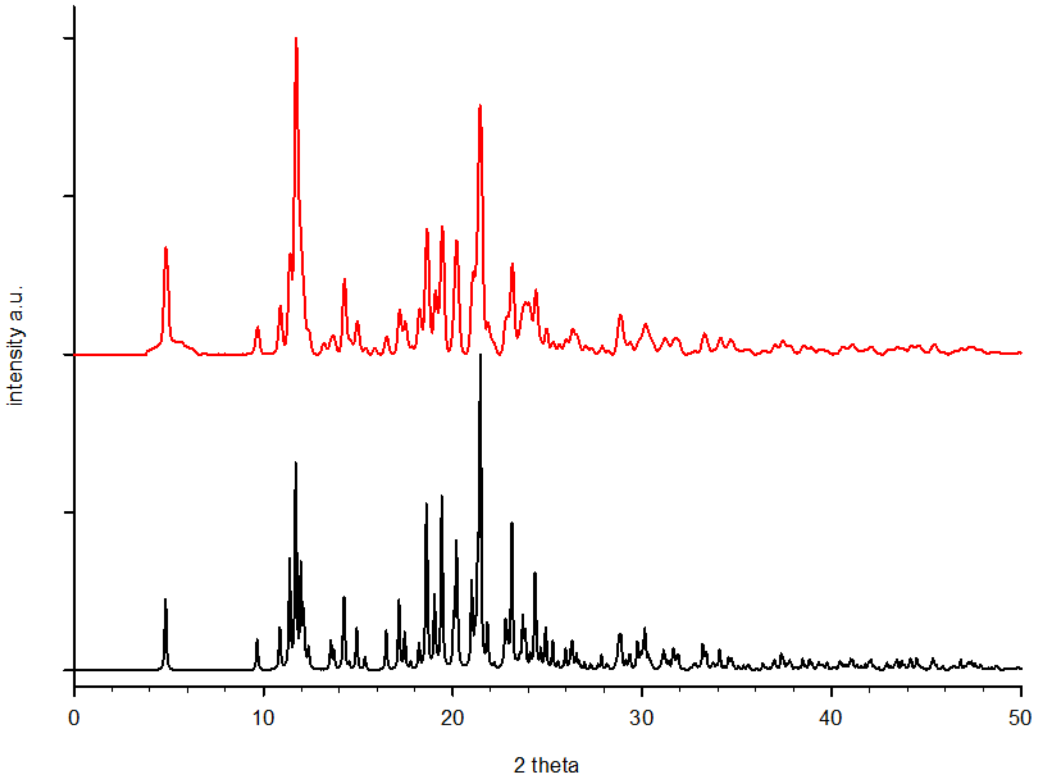

Figure SI-15. The recorded X-ray powder diffraction pattern (red) and predicted pattern based on single-crystal X-ray diffraction indices (black) of (**TrCOTyr**)$_2$·**NPHD.**

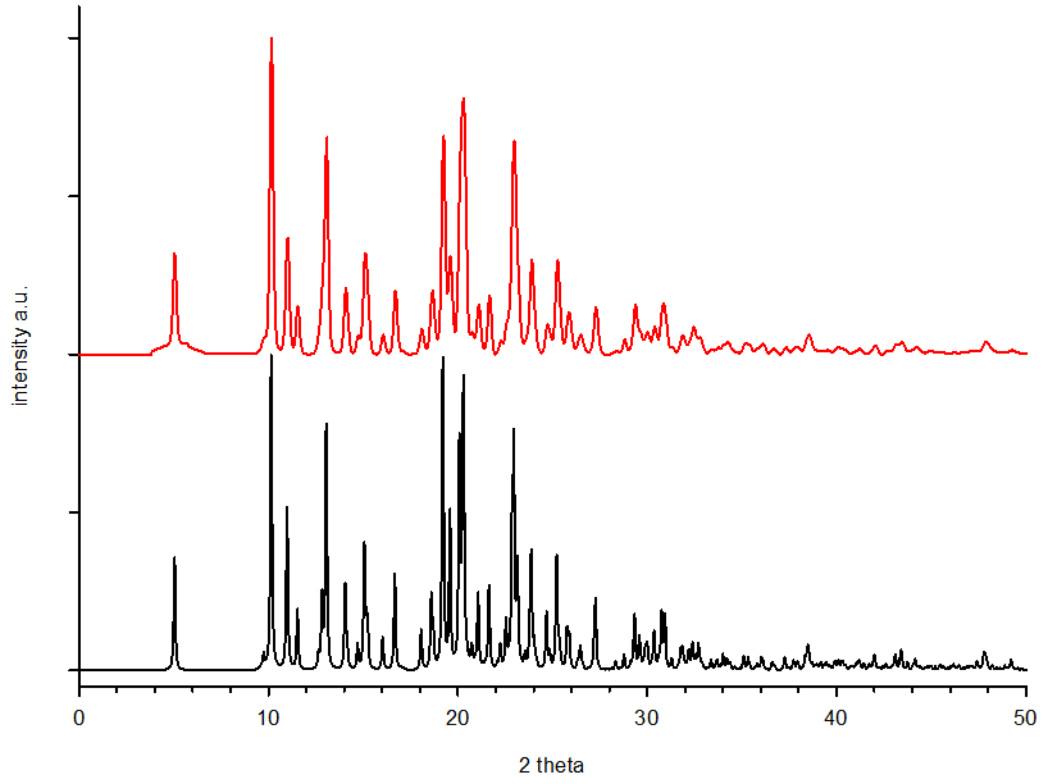

Figure SI-16. The recorded X-ray powder diffraction pattern (red) and predicted pattern based on single-crystal X-ray diffraction indices (black) of **TrCOTyr·QX**.

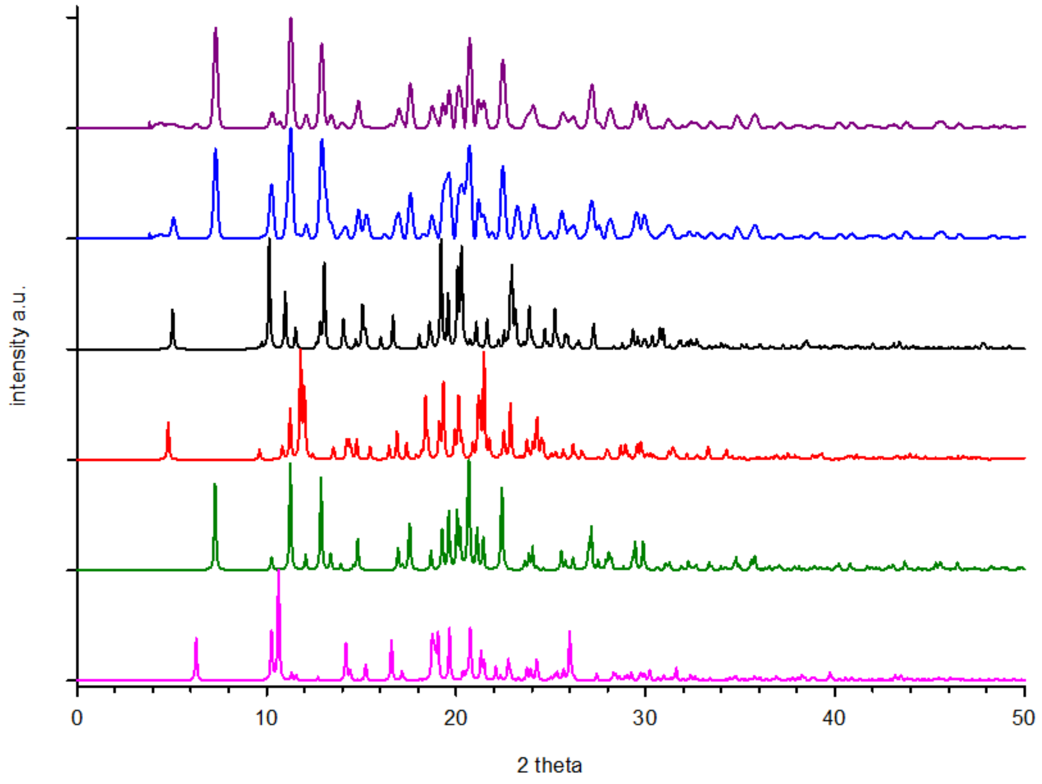

Figure SI-17. The recorded X-ray powder diffraction pattern of decomposition product of **TrCOTyr·QX**: sample heated to 130°C (blue) and sample heated to 180°C (violet); and predicted patterns based on single-crystal X-ray diffraction indices of: **TrCOTyr·QX** (black), (**TrCOTyr**)$_2$·**QX** (red), **TrCOTyr** polymorph I (green), *rac*-**TrCOTyr** (pink).

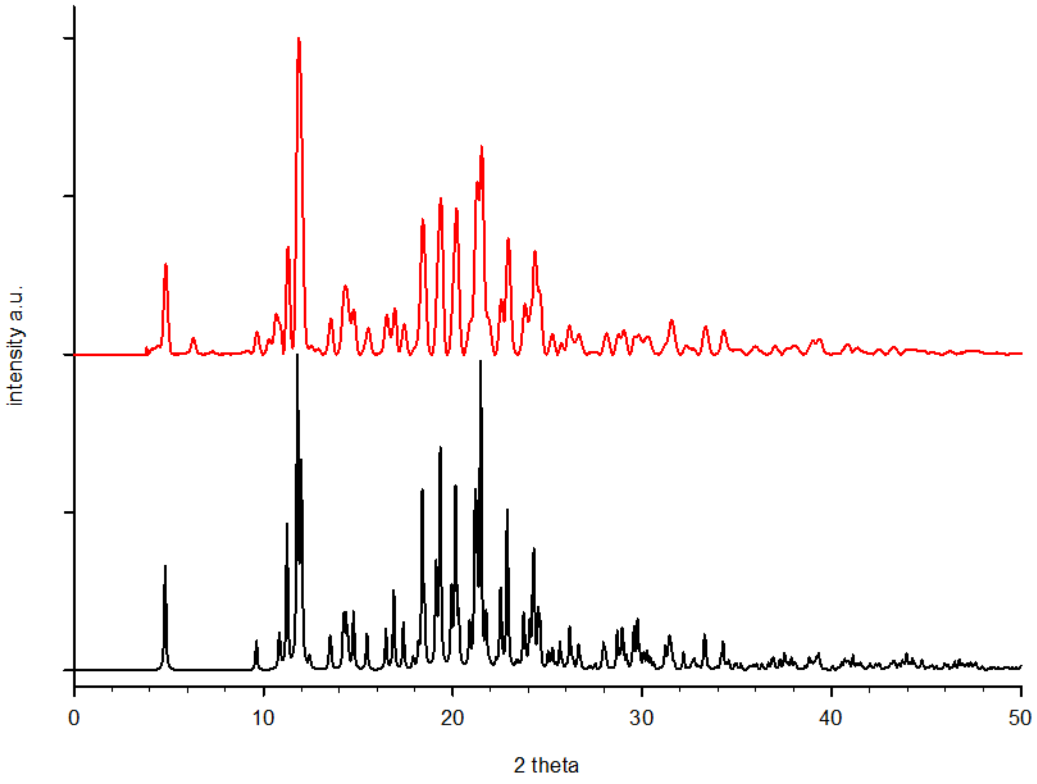

Figure SI-18. The recorded X-ray powder diffraction pattern (red) and predicted pattern based on single-crystal X-ray diffraction indices (black) of (**TrCOTyr**)$_2$·**QX**.

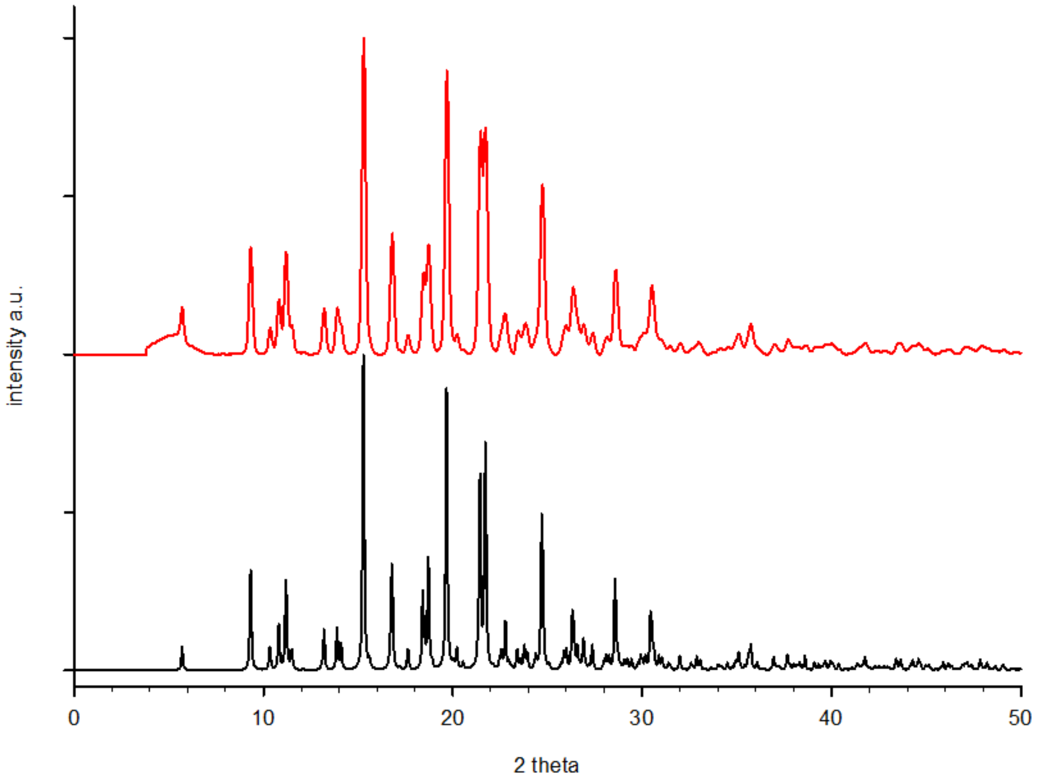

Figure SI-19. The recorded X-ray powder diffraction pattern (red) and predicted pattern based on single-crystal X-ray diffraction indices (black) of **(TrCOTyr)$_2$·BIPY**.

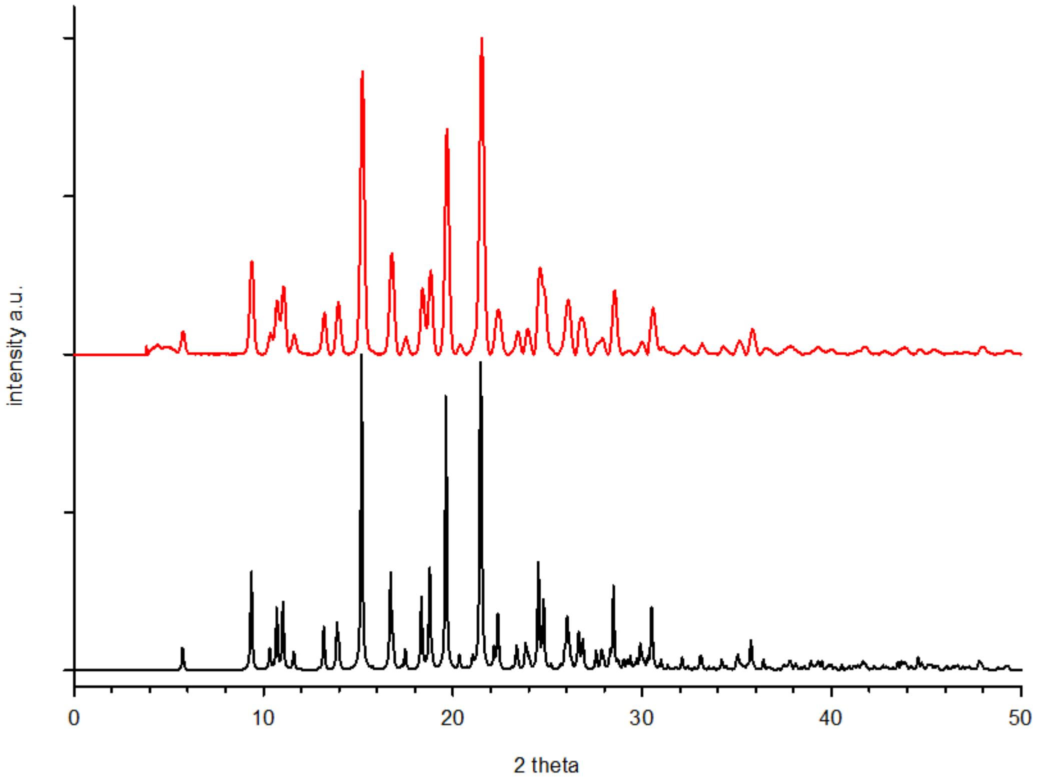

Figure SI-20. The recorded X-ray powder diffraction pattern (red) and predicted pattern based on single-crystal X-ray diffraction indices (black) of (*rac*-**TrCOTyr**)$_2$·**BIPY**.

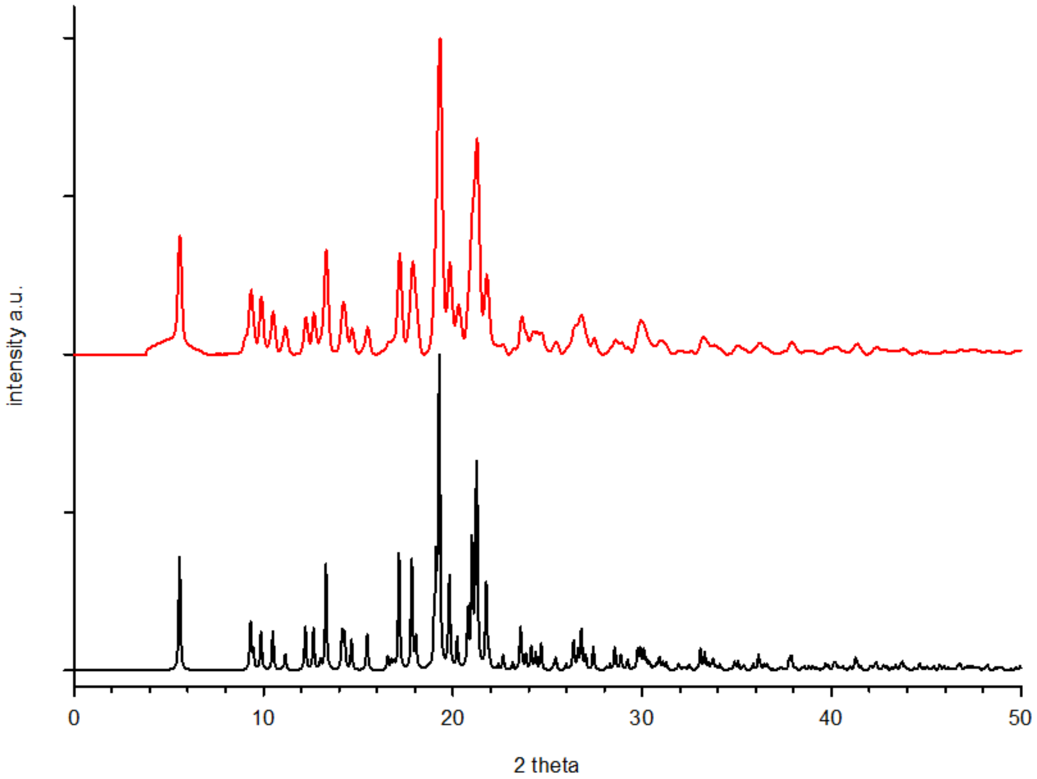

Figure SI-21. The recorded X-ray powder diffraction pattern (red) and predicted pattern based on single-crystal X-ray diffraction indices (black) of **TrCOTyr·DABCO**.

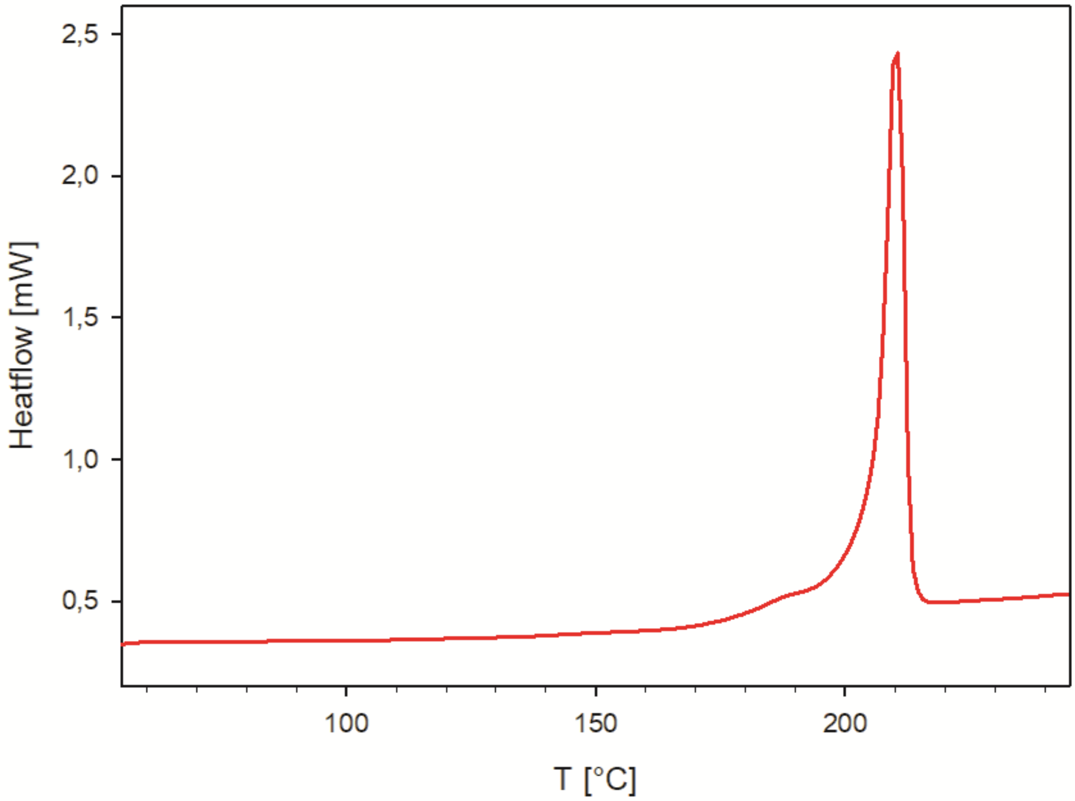

Figure SI-22. DSC curves of **TrCOTyr** (polymorph I).

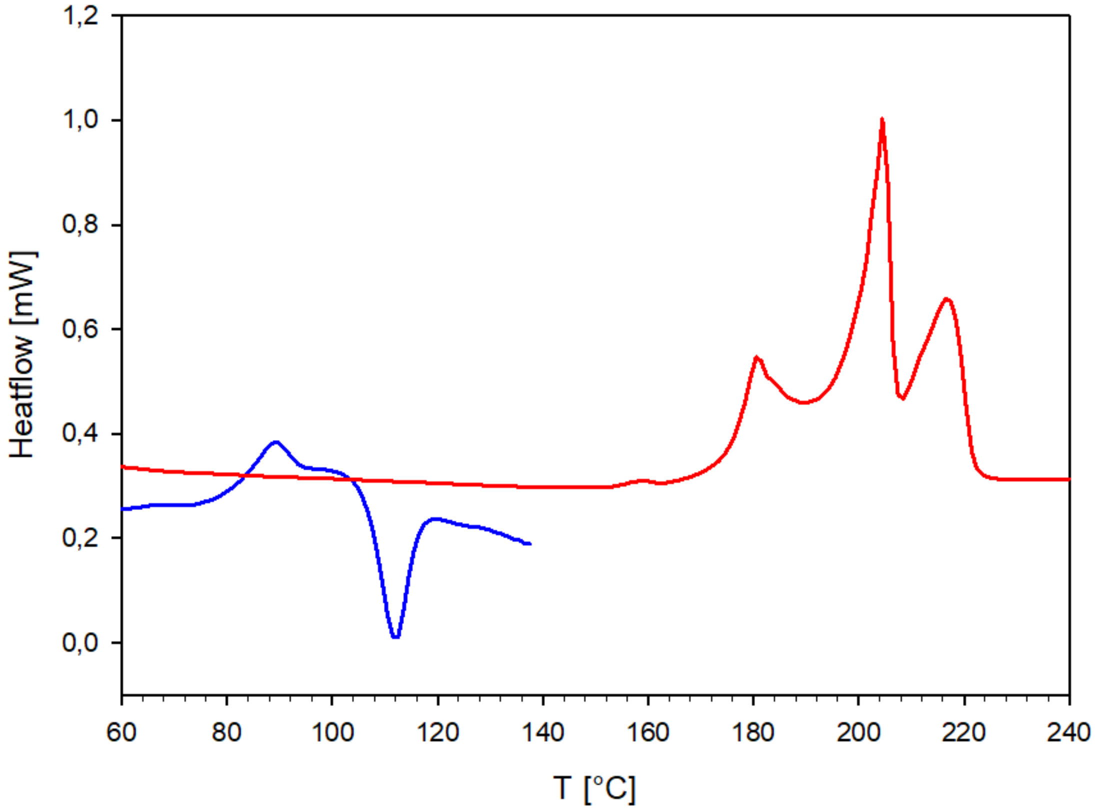

Figure SI-23. DSC curves of **TrCOTyr**·MeOH (blue color - the first heating cycle, red color - the second heating cycle).

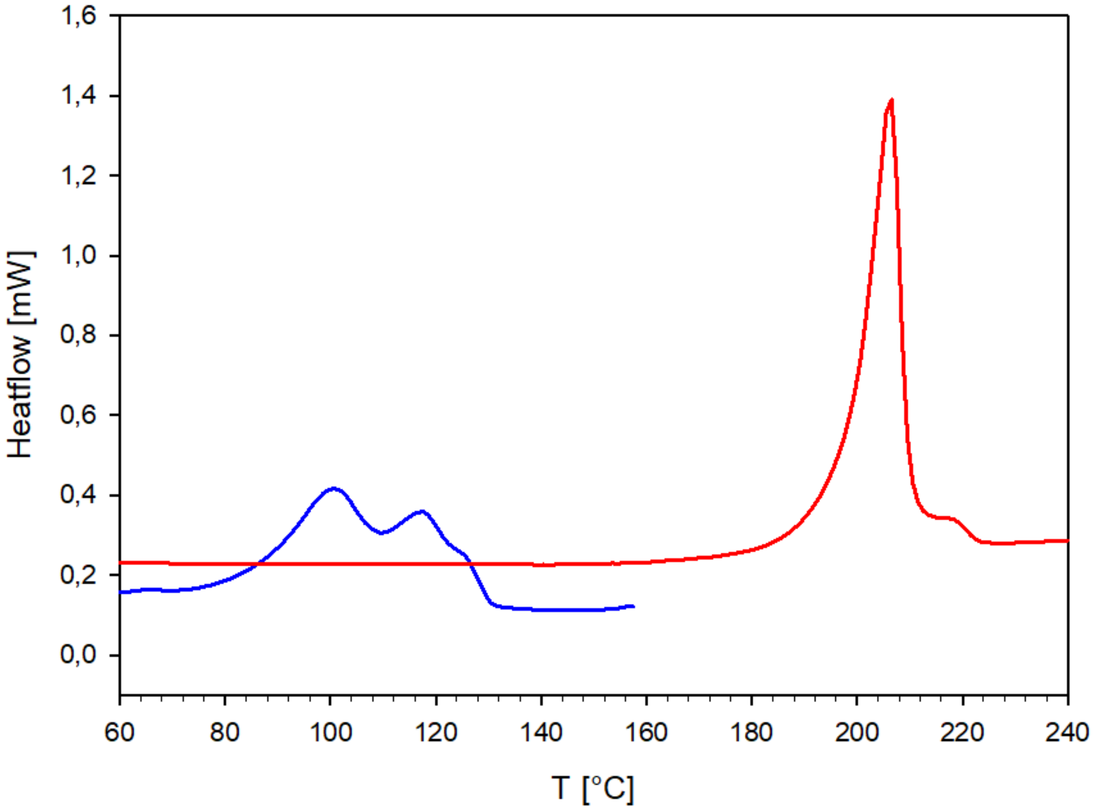

Figure SI-24. DSC curves of **TrCOTyr**·EtOH (blue color - the first heating cycle, red color - the second heating cycle).

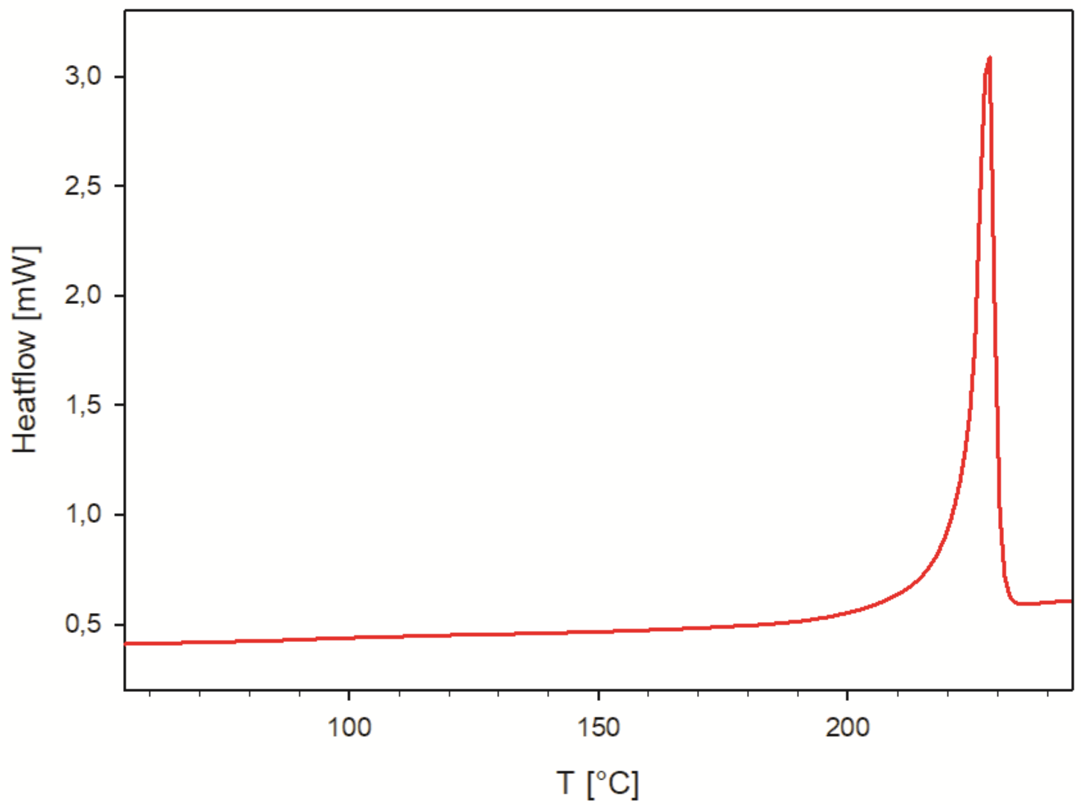

Figure SI-25. DSC curves of *rac*-**TrCOTyr**.

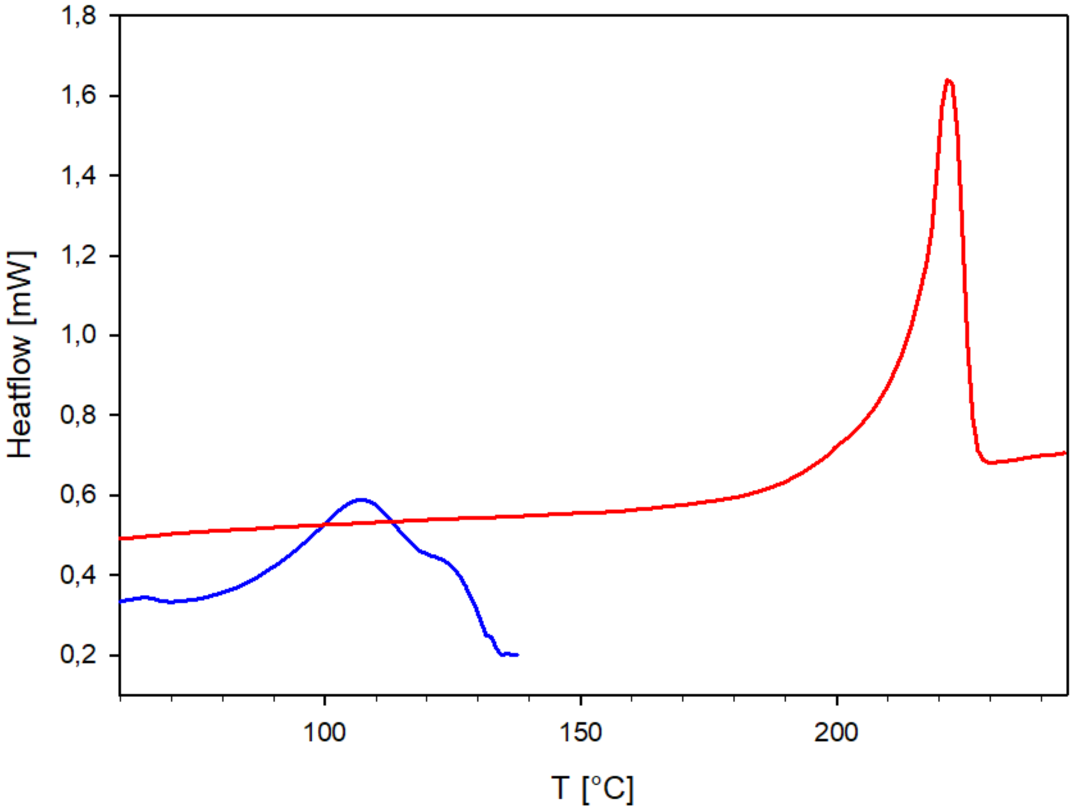

Figure SI-26. DSC curves of *rac*-**TrCOTyr**·MeOH (blue color - the first heating cycle, red color - the second heating cycle).

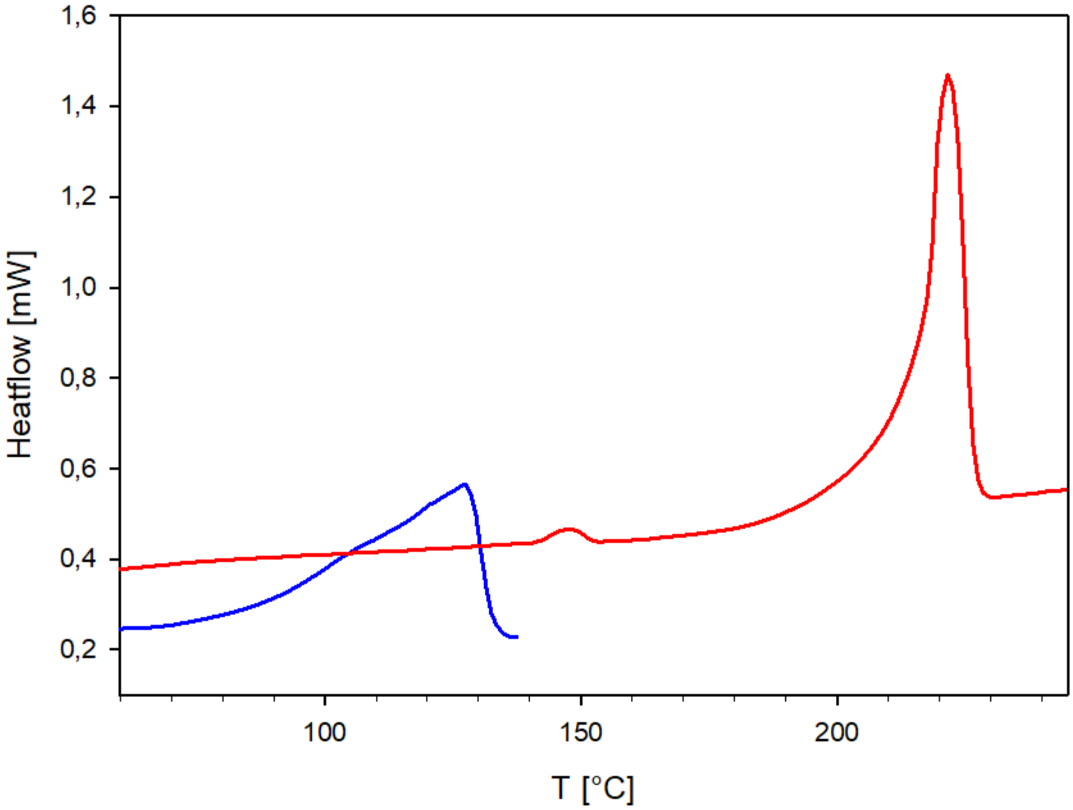

Figure SI-27. DSC curves of *rac*-**TrCOTyr**·EtOH (blue color - the first heating cycle, red color - the second heating cycle).

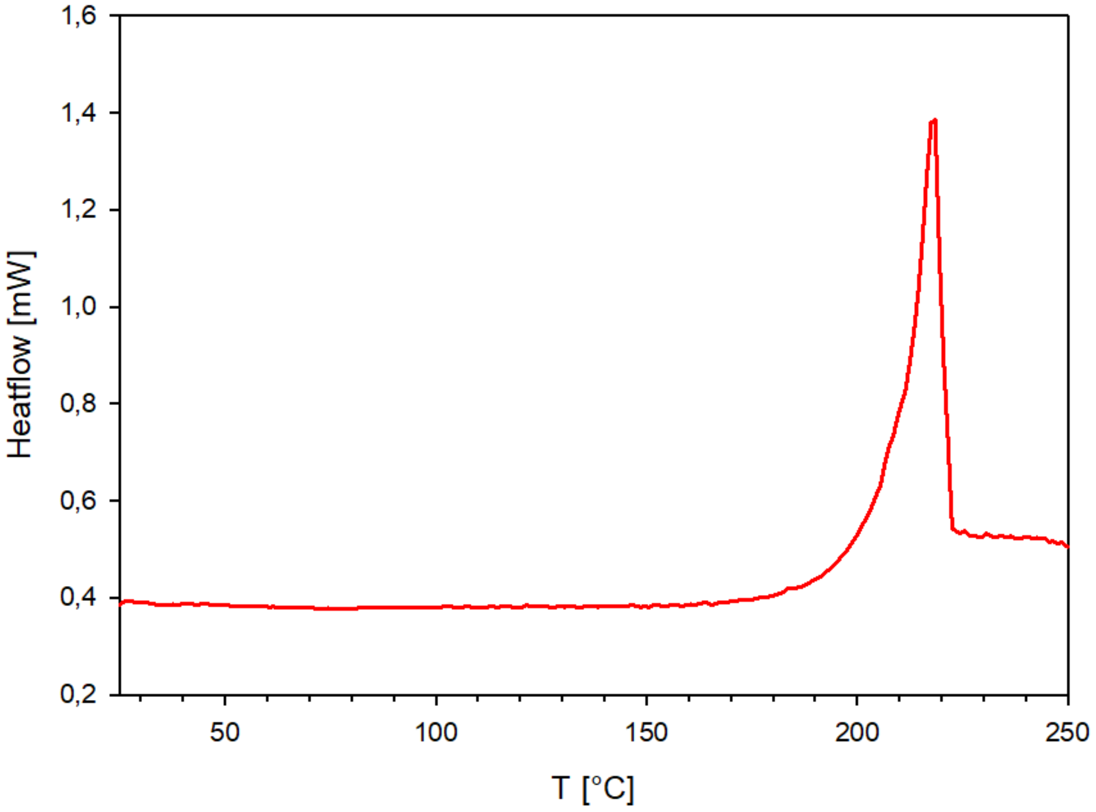

Figure SI-28. DSC curves of (**TrCOTyr**)$_2$·**NPHD**.

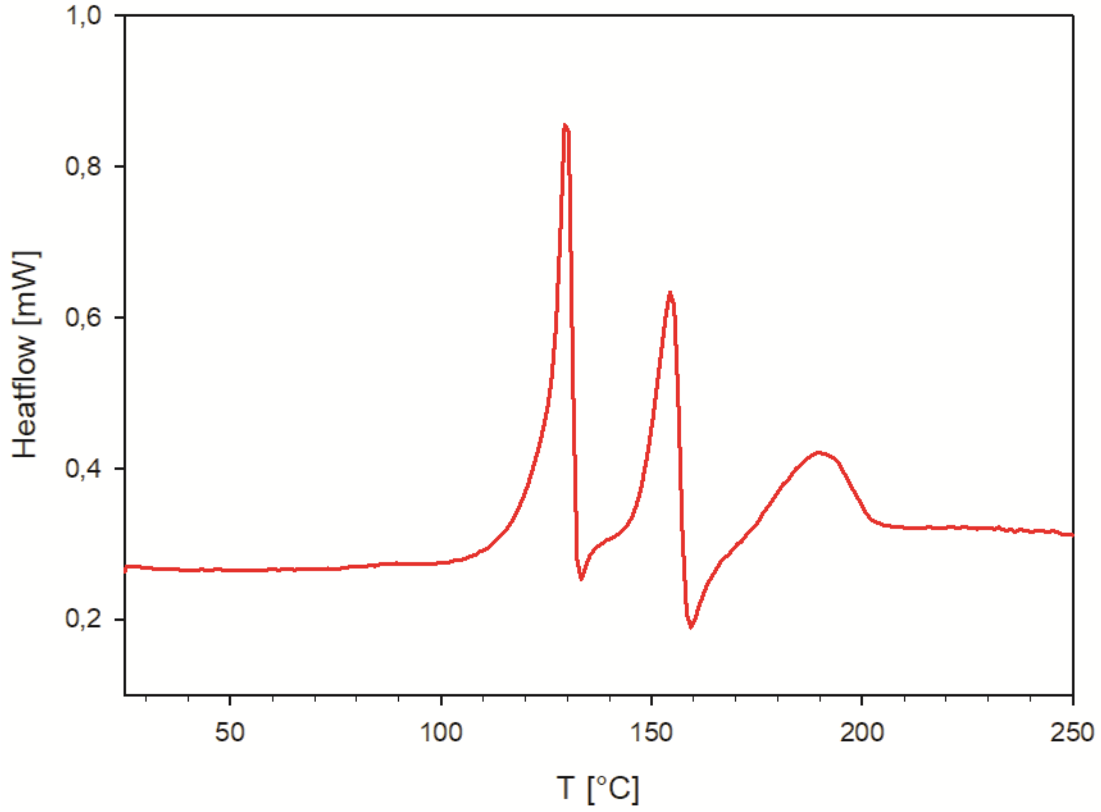

Figure SI-29. DSC curves of **TrCOTyr·QX**.

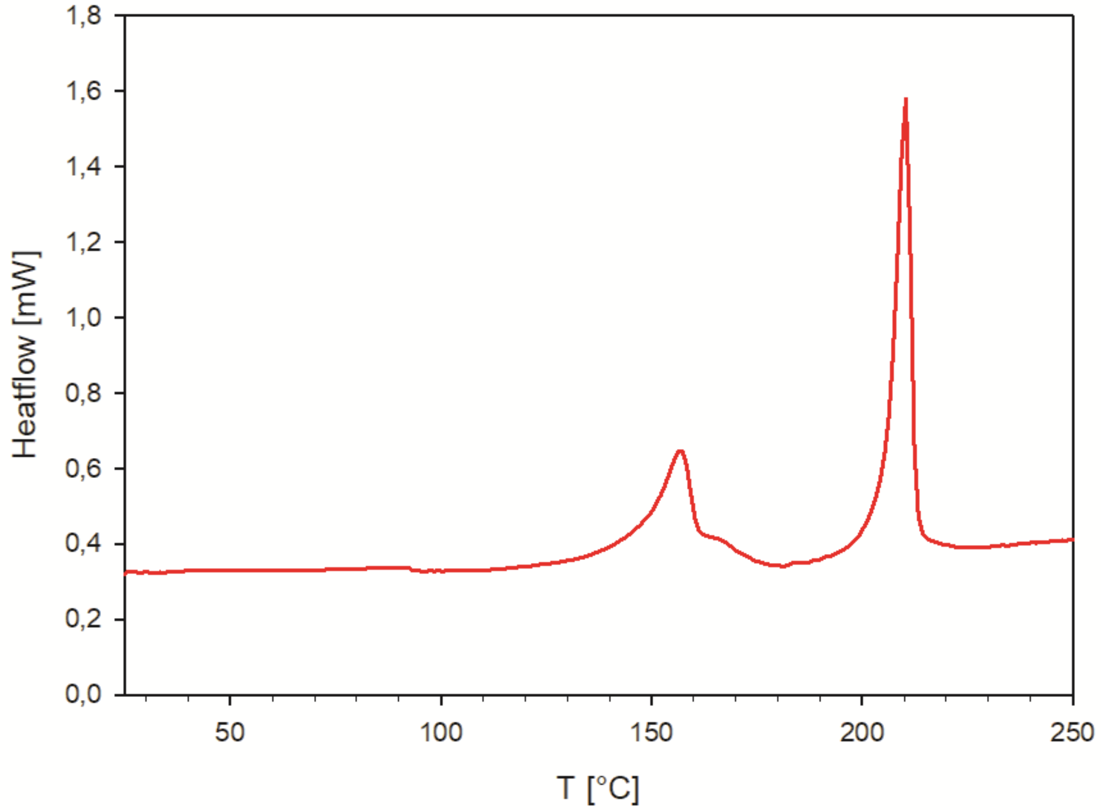

Figure SI-30. DSC curves of (**TrCOTyr**)$_2$·**QX**.

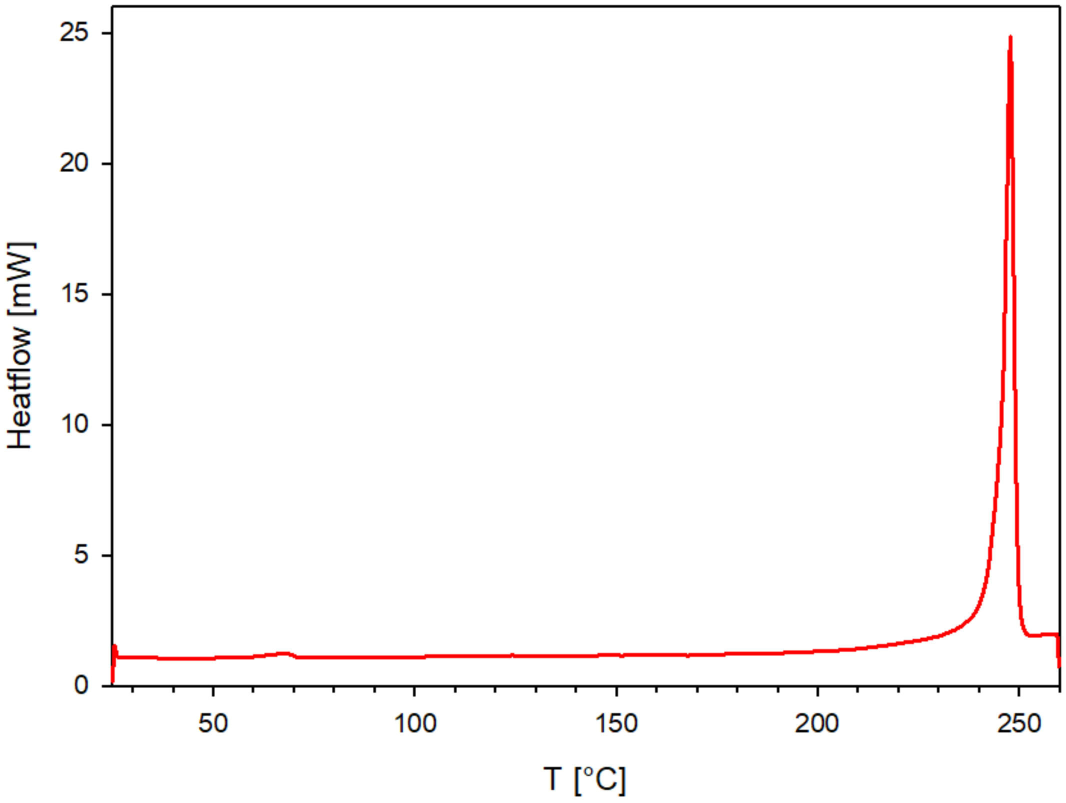

Figure SI-31. DSC curves of (**TrCOTyr**)$_2$·**BIPY**.

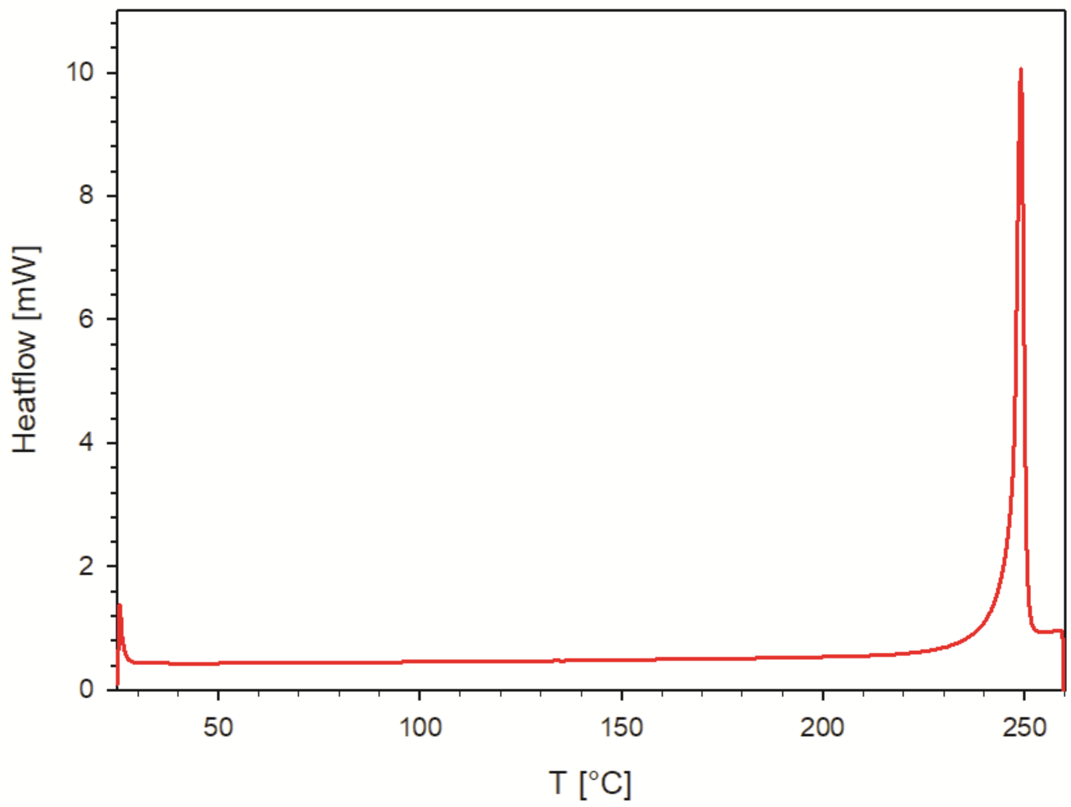

Figure SI-32. DSC curves of (*rac*-**TrCOTyr**)$_2$·**BIPY**.

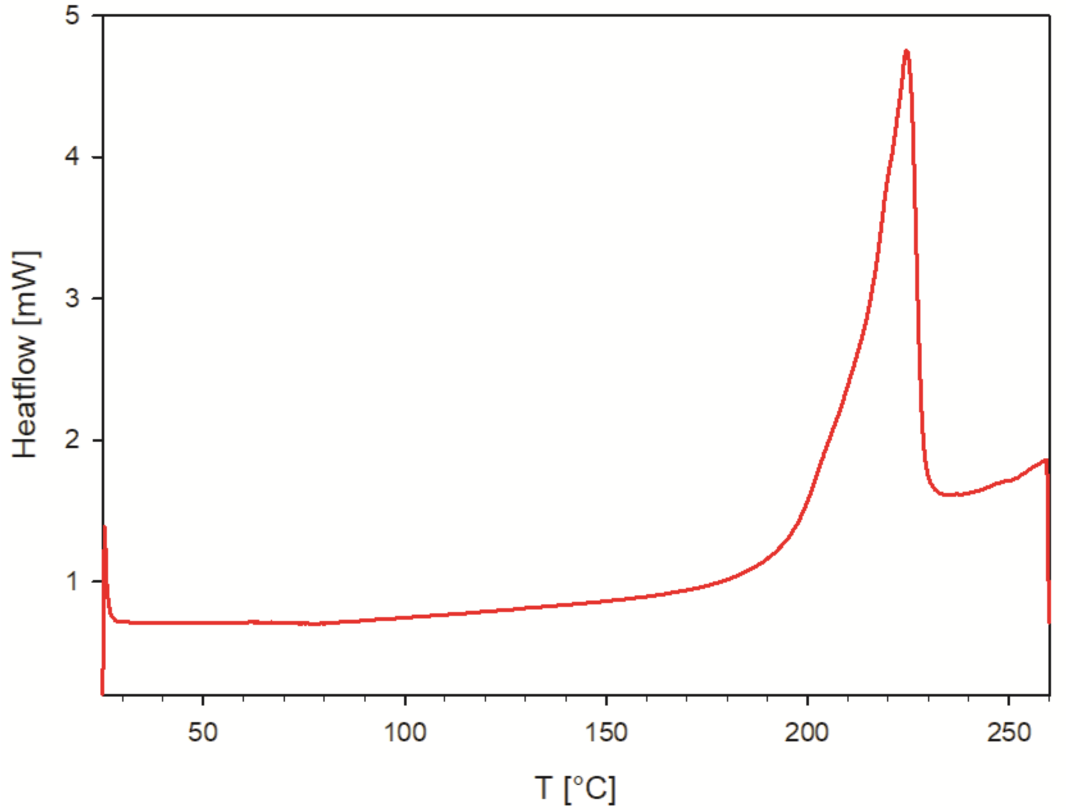

Figure SI-33. DSC curves of **TrCOTyr·DABCO**.
